# Eukaryotic Initiation Factor 4G Suppresses Nonsense-Mediated mRNA Decay by Two Genetically Separable Mechanisms

**DOI:** 10.1371/journal.pone.0104391

**Published:** 2014-08-22

**Authors:** Raphael Joncourt, Andrea B. Eberle, Simone C. Rufener, Oliver Mühlemann

**Affiliations:** 1 University of Bern, Department of Chemistry and Biochemistry, Bern, Switzerland; 2 Graduate School for Cellular and Biomedical Sciences, University of Bern, Bern, Switzerland; Institute of Molecular and Cell Biology, Singapore

## Abstract

Nonsense-mediated mRNA decay (NMD), which is best known for degrading mRNAs with premature termination codons (PTCs), is thought to be triggered by aberrant translation termination at stop codons located in an environment of the mRNP that is devoid of signals necessary for proper termination. In mammals, the cytoplasmic poly(A)-binding protein 1 (PABPC1) has been reported to promote correct termination and therewith antagonize NMD by interacting with the eukaryotic release factors 1 (eRF1) and 3 (eRF3). Using tethering assays in which proteins of interest are recruited as MS2 fusions to a NMD reporter transcript, we show that the three N-terminal RNA recognition motifs (RRMs) of PABPC1 are sufficient to antagonize NMD, while the eRF3-interacting C-terminal domain is dispensable. The RRM1-3 portion of PABPC1 interacts with eukaryotic initiation factor 4G (eIF4G) and tethering of eIF4G to the NMD reporter also suppresses NMD. We identified the interactions of the eIF4G N-terminus with PABPC1 and the eIF4G core domain with eIF3 as two genetically separable features that independently enable tethered eIF4G to inhibit NMD. Collectively, our results reveal a function of PABPC1, eIF4G and eIF3 in translation termination and NMD suppression, and they provide additional evidence for a tight coupling between translation termination and initiation.

## Introduction

The term “nonsense-mediated mRNA decay” (NMD) describes one or possibly several closely related posttranscriptional processes in eukaryotic cells leading to the degradation of mRNA molecules that fail to properly terminate translation (Kervestin and Jacobson, 2012; Schweingruber et al., 2013). NMD functions as a quality control system by targeting aberrant mRNAs harboring premature termination codons (PTCs), but at the same time it is also an important regulator of gene expression by affecting the abundance of 5–10% of all mRNAs in mammals [1]–[9]. Knockouts of genes encoding the NMD factors UPF1, UPF2 or SMG1 cause early embryonic lethality in mice [6], [10], [11], and knockdowns of UPF1, SMG1 or SMG6 are even lethal in cultured human cells (own unpublished observations), indicating that NMD is essential for viability of mammalian cells. Because approximately one third of all known disease-causing mutations in humans result in the production of PTC-containing mRNAs, NMD activity modulates the clinical manifestations of many of these genetic diseases, often to the benefit but sometimes to the disadvantage of the patients [12], [13]. There is therefore considerable medical and pharmaceutical interest in elucidating the molecular mechanism of NMD.

There is evidence that NMD is triggered by prolonged ribosome stalling at termination codons [14], [15]. Aberrant or maybe simply too slow translation termination is thought to allow the activation of the mRNA-bound UPF1, leading to the subsequent assembly of additional NMD factors, including the endonuclease SMG6 and/or the heterodimer SMG5-SMG7, which in turn recruits the CCR4-NOT deadenylase complex [16]–[19]. In addition, a link is provided between the NMD factors and the decapping complex by the human proline-rich nuclear receptor coregulatory protein 2 (PNRC2) [20].

This kinetic NMD model implies that proper translation termination depends on specific termination promoting signals. It is well documented that bringing the cytoplasmic poly(A)-binding protein (PABPC1 in human cells) into proximity of an NMD-eliciting termination codon suppresses NMD [15], [21]–[25]. How PABP antagonizes NMD is not yet understood, but the reported interaction of its C-terminal domain (PABC) with the eukaryotic release factor 3 (eRF3) [26]–[28] and the evidence for an interaction between eRF3 and UPF1 [23], [25], [29], [30] led to the model that a competition between UPF1 and PABP for interacting with eRF3 at the terminating ribosome determines whether or not NMD ensues [31]. Supporting this model, Singh and colleagues showed with recombinant proteins that PABPC1 efficiently antagonizes the interaction between eRF3 and UPF1 [25]. However, recent work with *S.cerevisiae* indicated that NMD activation is more complex and involves more than a simple competition between these factors [32].

Here, we analyzed the effect of different PABPC1 deletions or mutants on the stability of an NMD-subjected reporter mRNA using a tethering approach. Unexpectedly, an N-terminal fragment of PABPC1 comprising the first three RNA recognition motifs (RRMs) was sufficient for NMD suppression when tethered to the reporter transcript, whereas the eRF3-interacting PABC domain was dispensable. Since the eukaryotic translation initiation factor 4G (eIF4G) interacts with RRM1/RRM2 of PABPC1, we also tethered eIF4G downstream of the PTC in our NMD reporter and found that it suppressed NMD to the same extent as PABPC1. Subsequent mapping of the NMD-suppressing eIF4G domains revealed two apparently independent pathways of suppression. The N-terminus of eIF4G antagonized NMD through its interaction with PABPC1, and the eIF3-interacting core domain of eIF4G also suppressed NMD.

## Results

### PABPC1 in the vicinity of a PTC antagonizes NMD

**Figure 1:**
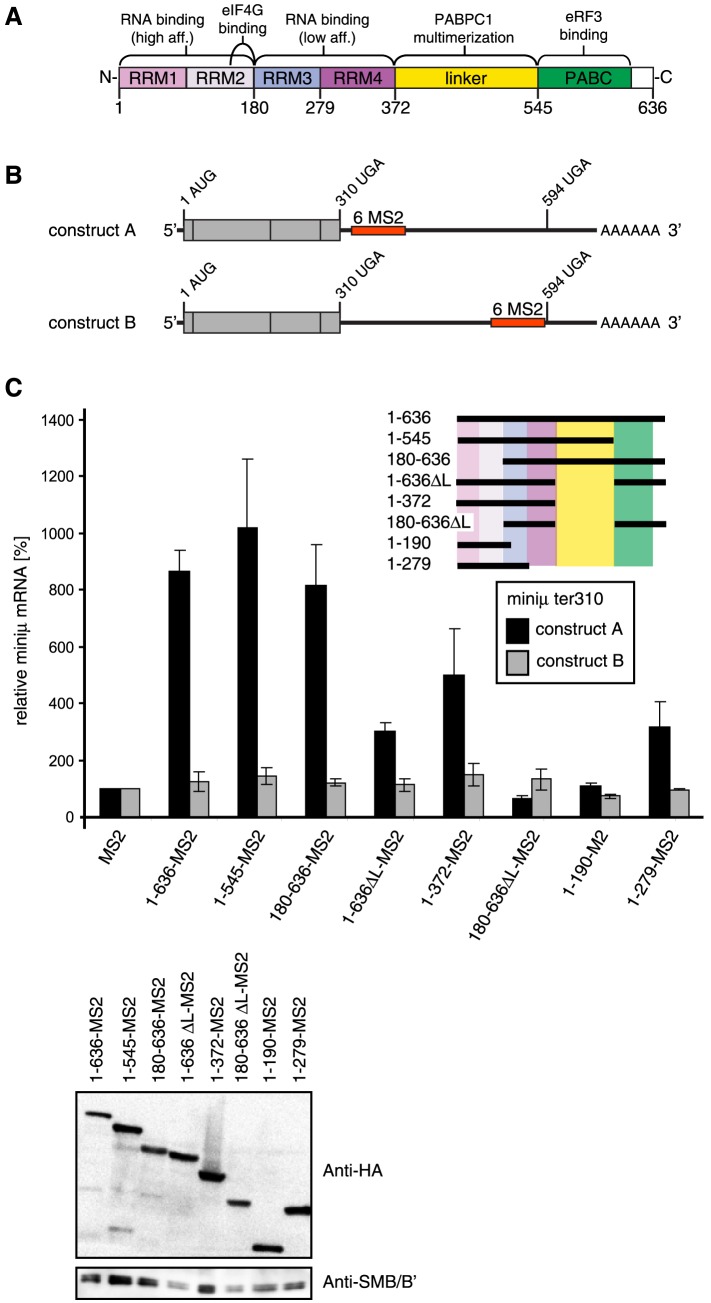
The C-terminal domain of PABPC1 is dispensable for inhibition of NMD, RRMs1-3 are sufficient, and RRMs1-2 are necessary. (A) Schematic representation of the eukaryotic cytoplasmic poly(A) binding protein (PABPC1). Domains are color-coded and assigned functions and interaction partners are depicted at the corresponding position above the scheme. Numbers below represent amino acid positions. (B) Schematic representation of the miniμ ter310 NMD reporter gene constructs A and B. The translation initiation codon (1 AUG), the premature translation termination codon at amino acid position 310 (310 UGA) and the position of the natural translation terminatinon codon (594 UGA) are shown. The position in the two constructs of the cassette with 6 MS2 binding sites (6 MS2) is depicted as a red box. (C) HeLa cells were transiently transfected with either the NMD reporter miniμ ter310 construct A or construct B, plasmids encoding the indicated PABPC1-MS2 fusion protein variants and a GPx1 expressing plasmid for normalization. 48 h post transfection, RNA was extracted and relative miniμ ter310 mRNA levels were determined by RT-qPCR. Miniμ mRNA levels were normalized to GPx1 mRNA levels and displayed relative to the sample with the MS2-HA protein (MS2, defined as 100%). The different PABPC1 mutants are shown schematically, using the same domain color-code as in (A). All are fused to the N-terminus of MS2 followed by a HA-tag at the C-terminus. Average values and standard deviations are shown of two independent experiments with two mRNA measurements each. A western blot showing the abundance of all PABPC1 fusion proteins is shown in the lower panel. The PABPC1-MS2 constructs were detected with an anti-HA antibody, endogenous SMB/B′ served as a loading control.

Tethering of proteins to NMD reporter transcripts is a powerful assay to identify proteins and domains thereof that are capable of promoting or antagonizing NMD. Using such tethering systems, PABPC1 was previously shown to suppress NMD when tethered to an NMD reporter mRNA in the vicinity of the PTC [15], [21]–[25] (**Figure 1**). PABPC1 is the main cytoplasmic PABP and consists of four non-identical RRMs followed by a linker region and the eRF3-binding PABC domain (**Figure 1A**). Each RRM contains two conserved RNPs (ribonucleoprotein domains) that are necessary and sufficient for binding RNA molecules in a wide range of specificities and affinities [33]. Poly(A) stretches are bound with high affinity by RRM1 and RRM2 of PABPC1 and with lower affinity by RRM3 and RRM4 [34]. A minimum of 12 nucleotides are needed for binding, but up to 25 nucleotides are covered upon saturation of long poly(A) [35]. RRM1 and RRM2 are also involved in the interaction to eIF4G and to the PABP-interacting proteins (PAIP1 and PAIP2) [36]–[38]. The interaction between the N-terminal part of eIF4G and RRM1/2 of PABPC1 results in a circularization of the mRNA [37], [39] that can influence translational processes. It is thought that the formation of this ‘closed loop’ structure of the mRNP is crucial for PABP's activities in promoting translation initiation and termination, recycling of ribosomes, and mRNA stability [40]–[44]. The four RRMs are followed by an unstructured, proline- and glutamine-rich linker region that is involved in the multimerisation of PABPC1 [35]. The C-terminal domain of PABPC1 (PABC, also known as MLLE) recruits several translation factors possessing the PABP-interacting motif 2 (PAM2) to the poly(A) tail of mRNAs [28], [38], [45]. Proteins with PAM2 include eRF3, eIF4B, PAIP1 and PAIP2 [26], [44], [46]–[49].

To map the domains of PABPC1 required for NMD suppression, different parts of PABPC1 were fused to the N-terminus of an HA-tagged MS2 coat protein and transiently expressed in HeLa cells, together with a miniμ ter310 NMD reporter gene containing six MS2 binding sites (6 MS2) located either approximately 50 nucleotides downstream of the ter310 codon (construct A) or several hundred nucleotides downstream as a control (construct B) (**Figure 1B**). For all experiments, the expression of the MS2 fusion proteins was monitored by SDS-PAGE and western blotting.

Confirming previous observations [21]–[25], tethering of full length PABPC1 (1–636-MS2) increased the abundance of construct A about 9-fold compared to tethering of MS2 alone, but led to no stabilization of the control construct B (**Figure 1C**), consistent with the proposed role of PABPC1 in antagonizing NMD and promoting correct translation termination.

### RRM1-2, but not the PABC domain, is required for NMD suppression

Next, we tested several deletion mutants of PABPC1 for their ability to increase the NMD reporter transcript in the tethering assay. Unexpectedly, deletion of the C-terminal part of PABPC1 (PABC; construct 1–545-MS2), which is crucial for its interaction with eRF3 [26], [27] and which was reported to be required for stabilization in previous tethering assays [24], increased the RNA level of reporter construct A as efficiently as full length PABPC1 (**Figure 1C**). A similar increase in NMD reporter RNA was observed by tethering of PABPC1 lacking RRM1-2 (180–636-MS2). Since PABPC1 was shown to multimerize through its linker domain (amino acids 372–545, **Figure 1A**) [50], we reasoned that the presence of this domain in our tethering assay might result in the co-recruitment of endogenous PABPC1 to the reporter transcript, thereby obscuring our mapping approach. Therefore, we deleted this linker domain from the subsequent PABPC1 deletion constructs. Deletion of the linker domain resulted in a loss of more than half of the stabilization effect seen with full length PABPC1, suggesting that full stabilization might indeed require multimeric PABP (**Figure 1C**, compare 1–636-MS2 with 1–636ΔL-MS2). Even in the context of the linker deletion, the PABC domain was dispensable for NMD inhibition: tethering of RRM1-4 (1–372-MS2) and RRM1-3 (1–279-MS2) of PABPC1 still increased the abundance of the NMD reporter construct A. On the other hand, deletion of the first 180 amino acid residues (180–636ΔL-MS2) resulted in the loss of construct A mRNA stabilization (**Figure 1C**). Taken together, these results indicate that the first two RRMs of PABPC1 are required and first three RRMs are sufficient to suppress NMD in our tethering assay.

### Interaction between PABPC1 and eIF4G provides a possible signal for NMD suppression

**Figure 2:**
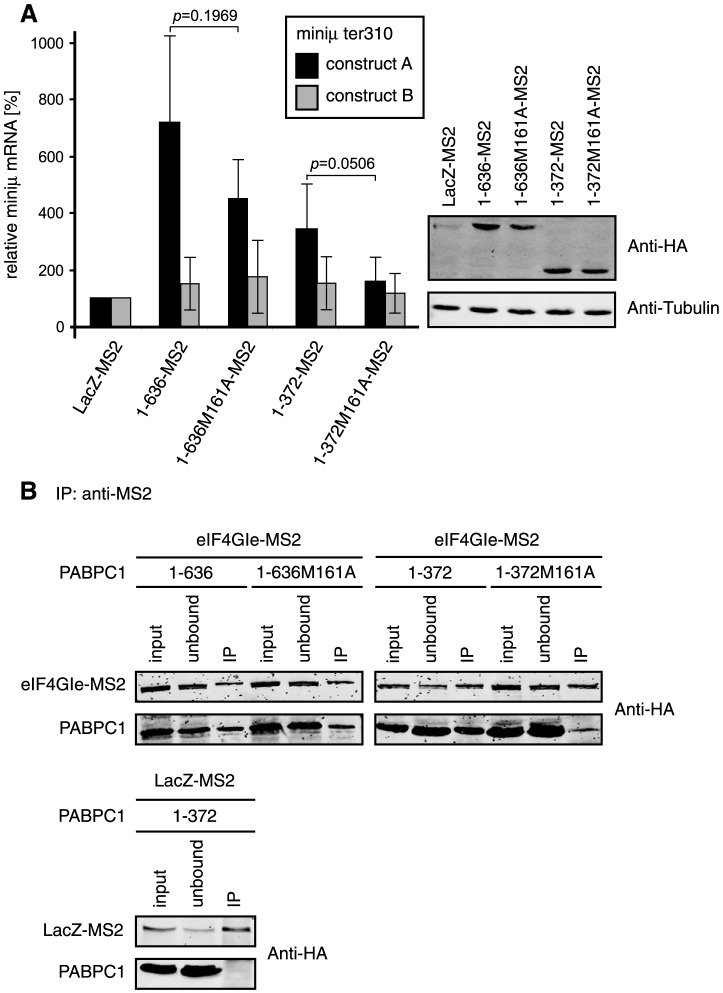
A PABPC1 mutation disturbing its association with eIF4G reduces its capacity to suppress NMD. (A) HeLa cells were transiently transfected with the NMD reporter miniμ ter310 construct A or construct B, plasmids encoding the indicated PABPC1-MS2 fusion protein variants and GPx1 as a normalizer. After RNA extraction and RT-qPCR, relative miniμ ter310 and GPx1 mRNA were measured and normalized as described in **Figure 1C**, except that the LacZ-MS2 samples were set as 100%. Full length PABPC1 (1–636-MS2) and a version comprising the first four RNA recognition motifs (1–372-MS2) with or without the M161A mutation are shown. All proteins are fused to the N-terminus of the MS2 moiety and contain a HA-tag at the C-terminus. Average values and standard deviations of at least three independent experiments are shown. The right panel shows a western blot using anti-HA and anti-tubulin antibodies to monitor the expression levels of the transfected MS2 fusion proteins and the endogenous tubulin as loading control, respectively. (B) To assess the effect of the M161A mutation in PABPC1 on the interaction with eIF4G, HEK 293T cells were transfected with plasmids encoding the eIF4GI isoform e fused to MS2 (eIF4Gle-MS2; see **Figure 4**) or LacZ-MS2 together with a plasmid encoding the indicated PABPC1 construct. All proteins were HA-tagged. 48 h post transfection, immunoprecipitations were performed using an anti-MS2 antibody and the association of the PABPC1 constructs with eIF4Gle was assessed by western blotting using an anti-HA antibody. Samples before (input) and the supernatant after (unbound) the immunoprecipitations represent 10% of the total material, and 50% of the immunoprecipitated material (IP) were loaded on the gel.

The described experiments imply that the first two RRMs are important for the increase of the reporter mRNA. Since PABPC1 interacts with eIF4GI through the second RRM [37], [39], [51], we wondered if eIF4GI might play a role in the observed effect on NMD reporters by tethered PABPC1. To investigate this, we introduced a mutation (M161A) into RRM2 that abolishes PABPC1's interaction to eIF4GI *in vitro* [41], [52]. We analyzed the effect of this mutation in the context of the full length PABPC1 (1–636-MS2) and the truncation comprising the four complete RRMs (1–372-MS2, **Figure 2A**). For this and all subsequent tethering experiments, we used a 70 kDa fragment of the bacterial β-galactosidase protein fused to MS2 (LacZ-MS2) as the negative control instead of the MS2 protein alone (**Figure 1C**), because we noticed during the course of our study that compared to no tether (mock transfection with a plasmid not expressing any MS2 fusion protein) or tethering of another unrelated protein (SLBP-MS2), LacZ-MS2 did not affect our reporter constructs, whereas MS2 alone consistently reduced the mRNA levels of the NMD reporter construct A (**Figure S1** and data not shown).

The eIF4G interaction mutants (1–636M161A-MS2 and 1–372M161A-MS2) had a reduced ability to suppress NMD of miniμ ter310 construct A compared to the corresponding non-mutated proteins (1–636-MS2, 1–372-MS2), with the lower NMD suppression capacity only being statistically significant for the shorter 1–372 construct (**Figure 2A**). Tethered PABPC1 1–372M161A-MS2A could also not suppress NMD on another reporter construct consisting of the TCRβopen reading frame and a PTC at amino acid position 68, followed by six MS2 binding sites in close proximity (TCRβ ter68 construct A) or further downstream (TCRβ ter68 construct B; **Figure S2**; see also [22]). On both reporter transcripts, we observed with the RRM1-4 constructs that the mutant (1–372M161A-MS2) lost its ability to increase the reporter mRNA levels, suggesting that NMD inhibition mediated by the tethered RRM1-4 PABPC1 requires interaction with eIF4G.

Next we compared the capacity of M161A mutants and non-mutated PABPC1 versions for binding to eIF4G by co-immunoprecipitation assays (**Figure 2B**). Initial attempts to co-immunoprecipitate endogenous eIF4G with PAPBC1-MS2-HA constructs failed, possibly because only a very small fraction of the highly expressed recombinant PABPC1 assembled with eIF4G. Notably, this problem was also reported by Imataka and colleagues [37]. Therefore, we decided to exogenously express eIF4GI (isoform e, fused to MS2-HA) together with HA-tagged PABPC1 constructs (lacking the MS2 coat protein), followed by immunoprecipitation of eIF4GIe-MS2-HA using an anti-MS2 antibody and probing for associated PABPC1 using an anti-HA antibody. Hence, eIF4GIe-MS2 was co-transfected with the full length PABPC1, with (1–636M161A) or without (1–636) the mutation, or with the RRM1-4 constructs with (1–372M161A) or without (1–372) the mutation. As a control, LacZ-MS2 was immunoprecipitated from cells co-expressing PABPC1 1–372. No PABPC1 co-purified with LacZ-MS2, confirming the specificity of our assay (**Figure 2B**, lower panel). Similar amounts of full length PABPC1 co-immunoprecipitated with eIF4GIe-MS2, irrespectively whether PABPC1 carried the M161A mutation or not (**Figure 2B**, upper left panel). The presence of the linker domain in the full length PABPC1 constructs probably enables the M161A mutant to multimerize with endogenous PABPC1, which in turn interacts with eIF4GIe (see below). Consistent with this explanation, the M161A mutation led to a reduced association of truncated PABPC1 (1–372) with eIF4GIe-MS2 (**Figure 2B**, upper right panel), indicating that the *in vitro* identified M161 residue is only critical for the interaction with eIF4G *in vivo* under conditions that prevent PABPC1 multimerization. Despite of the complication due to PABPC1 multimerization, collectively these results suggest a role of eIF4G in antagonizing NMD.

### Depletion of eIF4GI reduces NMD suppression by PABPC1

**Figure 3:**
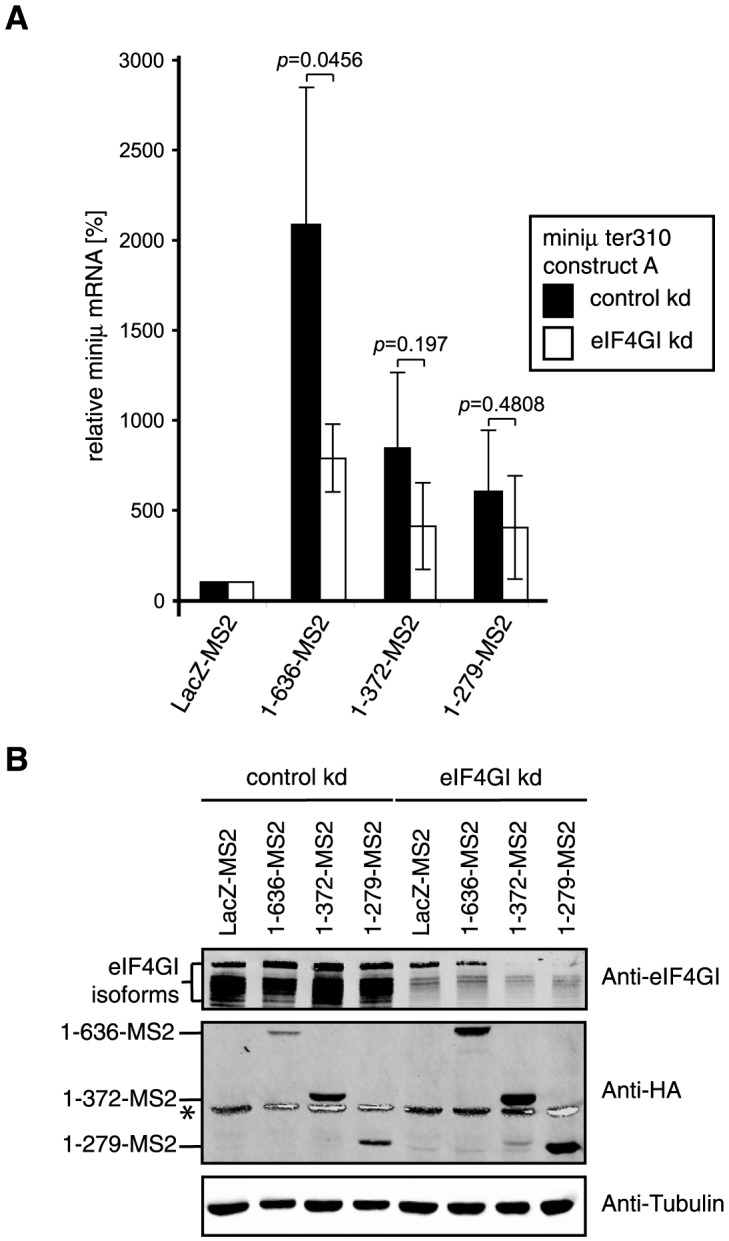
Depletion of eIF4GI diminishes the stabilization conferred by the full length PABPC1. (A) HeLa cells were transiently transfected with the NMD reporter miniμ ter310 construct A, a plasmid encoding the indicated MS2 fusion protein and one encoding GFP for normalization. In addition, the cells were transfected with a plasmid expressing an shRNA with a scrambled sequence (control kd) or with a sequence directed against the eIF4Gl mRNA (eIF4Gl kd). Total mRNA was extracted 96 h after transfection, relative miniμ and GFP mRNA levels were measured by RT-qPCR, miniμ mRNA was normalized to GFP mRNA and displayed relative to the LacZ-MS2 samples (set to 100%). Full length PABPC1 (1–636-MS2), truncations consisting of the first four RNA recognition motifs (1–372-MS2) or the first three RNA recognition motifs (1–279-MS2) were expressed, each with a C-terminal MS2-HA moiety. Average values and standard deviations of three independent experiments are shown. (B) Western blot to monitor relative protein levels using antibodies against eIF4GI, the HA-tag and endogenous tubulin. Endogenous eIF4GI was surveyed to assess the efficacy of the knockdowns, anti-HA allowed detection of the MS2 fusion proteins, and anti-tubulin served as a control for sample loading. * depicts the high intensity tubulin signal bleeding into the HA-channel of the infrared imaging system.

To further investigate the apparent role of eIF4GI in PABPC1-mediated NMD suppression, we performed our PABPC1 tethering assay in cells depleted for eIF4GI (**Figure 3**). Knockdown of eIF4GI was achieved by expressing an shRNA directed against eIF4GI mRNA (**Figure 3**, eIF4GI kd). Expression of an shRNA that is predicted to not target any human mRNA served as a control (control kd). The efficacy of the eIF4GI knockdown was monitored by western blotting (**Figure 3B**, Anti-eIF4GI). The tethering assay was performed with LacZ-MS2 (as the control), full length PABPC1 (1–636-MS2) and the two truncations still able to stabilize the NMD reporter (1–372-MS2 and 1–279-MS2).

In comparison to the previous experiments, the reporter mRNA increased much more by tethering the different PABPC1 variants (**Figure 3A**). For example, 1–636-MS2 increased the reporter about 20 fold, compared to 7–8 fold previously. In the knockdown experiments, the reporter mRNA and the tethered proteins are expressed in the cells for a longer time than in the standard tethering experiments, and we speculate that this prolonged time window allowed the stabilized NMD reporter mRNA to accumulate to higher levels. More importantly, in the eIF4GI-depleted cells the reporter increased mRNA level caused by tethered full length PABPC1 (1–636-MS2) was significantly reduced relative to the control knockdown. A less pronounced and statistically not significant reduction was also observed with the truncations containing all four RRMs (1–372-MS2) or RRMs1-3 (1–279-MS2). Depletion of eIF4GI is known to affect multiple cellular processes including protein synthesis [53]. Since NMD depends on translation, one might have expected eIF4GI depletion to result in a further stabilization of the NMD reporter transcript. Contrary to this, the observed reduction in stabilization of the reporter mRNA levels in eIF4GI-depleted cells provides further evidence for a role of eIF4GI in suppression of NMD by tethered PABPC1.

### Tethered eIF4GI isoforms capable of interacting with PABPC1 suppress NMD

**Figure 4:**
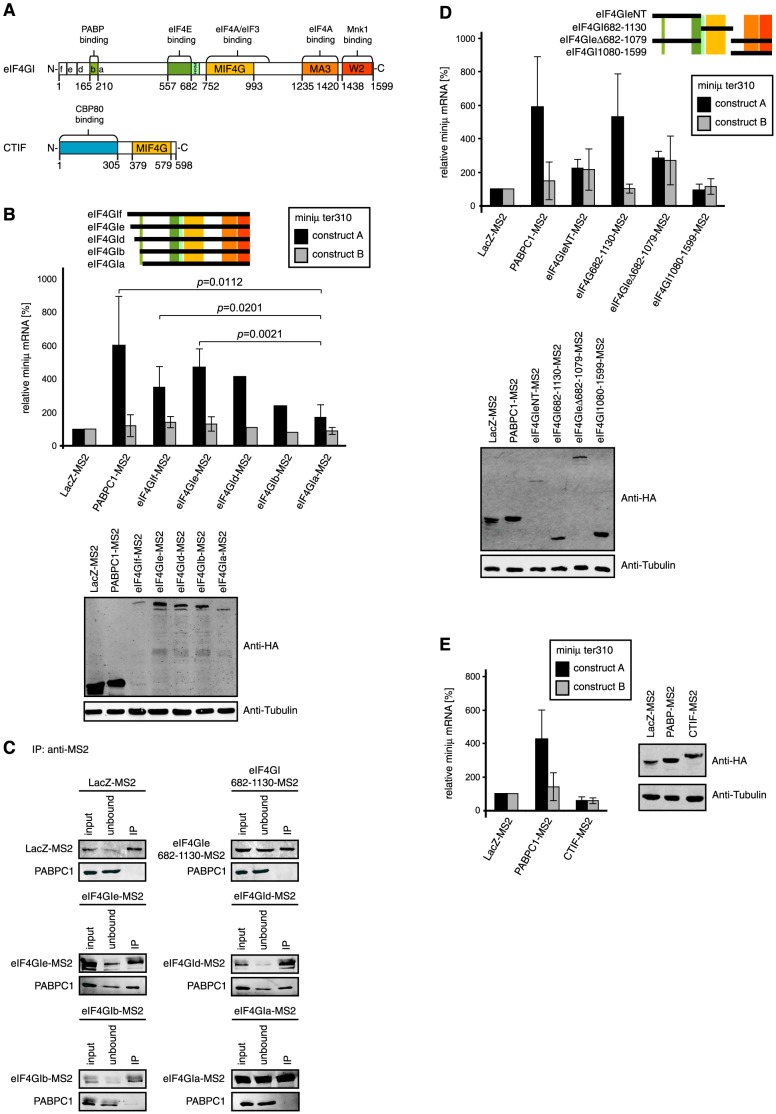
Tethering of the core domain of eIF4GI inhibits NMD to a similar extent as PABPC1. (A) Schematic representations of the eukaryotic initiation factor 4GI (eIF4GI; adapted from [53], [80]) and the CBP80/20-dependent translation initiation factor (CTIF; adapted from [57]). Functional domains are color-coded and labeled (RRM, RNA recognition motif; MIF4G, middle domain of eIF4G and HEAT-1 domain; MA3, HEAT-2 domain and MA3 region; W2, HEAT-3 and W2 domain). The letters (f, e, d, b, a) indicate the different N-termini of these five eIF4GI isoforms. Numbers below represent the respective amino acid positions, with 1 depicting the N-terminus of the longest eIF4GI isoform, eIF4Gf. Isoform c, which is 1 amino acid shorter than isoform d, is not shown. (B) Tethering of the different eIF4GI isoforms shown schematically on the right (compare with **Figure 4A**). HeLa cells were transiently transfected with the NMD reporter miniμ ter310 construct A or construct B, a plasmid encoding the indicated eIF4GI-MS2 fusion protein and one encoding GPx1 for normalization. The assay was performed as in **Figure 1C**. Average miniμ mRNA levels and standard deviations of at least four independent experiments, normalized to GPx1 mRNA and displayed relative to the LacZ-MS2 samples are shown, except for eIF4Gld and eIF4Glb where the results of only one experiment are shown. Full length PABPC1-MS2 corresponds to 1–636-MS2 in previous Figures. All MS2 fusion proteins contain a C-terminal HA-tag. The lower panel represents a western blot probed with anti-HA and anti-Tubulin antibodies to assess the relative expression of the MS2-fusion proteins and of endogenous tubulin used as a loading control, respectively. (C) To test for association of endogenous PABPC1 with eIF4GI variants, plasmids encoding the indicated eIF4GI isoforms or the eIF4GI core domain (eIF4GI682-1130-MS2) fused to MS2-HA were transfected into HEK 293T cells. After 48 h, the cell extracts were subjected to immunoprecipitation using an anti-MS2 antibody. LacZ-MS2 expressing cells served as a specificity control. The MS2 fusion proteins were detected with an antibody against the HA-tag (upper panels) and endogenous PABPC1 was detected with the mouse anti-PABPC1 10E10 antibody (lower panels). 10% of the cell extracts before (input) and the supernatant after (unbound) the immunoprecipitations, and 50% of the immunoprecipitated material (IP) were loaded on the gel. (D) Tethering assay as in **Figure 1C**, but with the eIF4GI deletion mutants depicted schematically on the right. All MS2 fusion proteins also contain a C-terminal HA-tag. Lower panel, western blot as in **Figure 4B**. (E) Tethering assay as in **Figure 1C**, but with CTIF-MS2. Average values and standard deviations of three independent experiments are shown. The western blot shown on the right side was done as in **Figure 4B**.

Next we wanted to test whether direct tethering of eIF4GI to our NMD reporter would also lead to its stabilization. Six isoforms of eIF4GI are known that differ in the length of the N-terminus of the protein, with the shortest isoform being called a and the longest one f (**Figure 4A**). Isoforms c and d differ in only one amino acid and are therefore usually not distinguished [53]. We fused to the C-terminus of the eIF4GI isoforms f, e, d, b, and a the MS2 coat protein and transiently transfected these plasmids into HeLa cells together with either the miniμ ter310 NMD reporter gene construct A or B (**Figure 4B**). Consistent with previous reports [53], we noticed that eIF4GI was difficult to over-express, especially the longest isoform eIF4GIf (**Figure 4B**, lower panel). Relative to the control (LacZ-MS2), reporter mRNA levels of construct A were elevated about 4-fold by tethering of isoforms f, e and d, which is similar as tethering of PABPC1-MS2 (**Figure 4B**, upper panel). In contrast, the two shortest isoforms (eIF4GIb and eIF4GIa) were less efficient in increasing the reporter transcript levels.

Interestingly, the drop in the capacity to increase the reporter mRNA level correlates with the potential loss of the ability to interact with PABPC1. The shortest isoform, eIF4GIa, lacks the PABP-binding region (amino acids 165–210, **Figure 4A**), and in eIF4GIb the proximity of the N-terminus might disturb the interaction of this amino acid stretch to PABPC1. To experimentally test the ability of the different eIF4GI isoforms for interacting with PABPC1, we performed immunoprecipitation experiments (**Figure 4C**). Different eIF4GI variants were expressed as MS2 fusion proteins in HEK 293T cells and subsequently immunoprecipitated with an antibody against the MS2 coat protein. The longest isoform (eIF4GIf) had to be omitted from this analysis due to its low expression level (**Figure 4B**). Using an anti-PABPC1 antibody, we then probed for co-immunoprecipitated endogenous PABPC1. Confirming the specificity of the immunoprecipitations, PABPC1 did not co-purify with LacZ-MS2. As predicted, the two longer isoforms e and d, which both contain the PABP-binding domain and were active in the tethering assay, both co-immunoprecipitated PABPC1. In contrast and as suspected, PABPC1 interaction with the two short eIF4GI isoforms was impaired with almost no PABPC1 detectable in the immunoprecipitate of eIF4GIb and none in the case of eIF4GIa (**Figure 4C**). Thus, consistent with our results from tethering PABPC1 fragments, this identifies the interaction between PABPC1 and eIF4G as a crucial element for preventing NMD.

### The core domain of eIF4GI inhibits NMD in a similar manner as PABPC1

To define additional parts of eIF4GI important for suppressing NMD in our tethering assay, we divided eIF4GI roughly into three parts (**Figure 4A and D**). For constructs containing the N-terminus we used isoform eIF4GIe, because eIF4GIf failed to express consistently to sufficiently high levels. The first fusion protein (eIF4GIeNT-MS2) consists of the N-terminal third of eIF4GIe encompassing the PABP and eIF4E interaction domains (corresponding to amino acids 41–681 in eIF4GIf). The second construct (eIF4GI682-1130-MS2) comprises the central MIF4G (middle domain of eIF4GI) domain, and the third fragment encompasses the C-terminal part of eIF4GI (eIF4GI1080-1599-MS2), which includes the two C-terminal HEAT repeat domains. Additionally, we also tested an eIF4GIe construct missing the central MIF4G domain (eIF4GIeΔ682-1079-MS2). As before, one of these MS2 fusion protein-encoding plasmids was co-transfected into HeLa cells together with the miniμ ter310 NMD reporter plasmid, either encoding construct A or B. LacZ-MS2 and PABPC1-MS2 served again as negative and positive controls, respectively (**Figure 4D**). The N-terminal portion of eIF4GI (eIF4GIeNT-MS2) was not able to increase the mRNA level of reporter construct A >2 fold relative to LacZ-MS2 and the same effect was observed when the MS2 binding sites were located distant from the PTC (construct B). This result was unexpected, because eIF4GIeNT-MS2 contains the PABP interaction domain. A caveat here is that compared to the other MS2 fusion proteins, eIF4GIeNT-MS2 expression was low (**Figure 4D**, lower panel).

Interestingly, tethering of the eIF4GI core domain (eIF4GI682-1130-MS2) increased the reporter transcript to a similar extent as full length PABPC1, even though its abundance was weaker than that of PABPC1 (**Figure 4D**). Importantly, this reporter mRNA accumulation mediated by the eIF4GI core domain seems to function independently of PABPC1, based on the absence of PABPC1 in the immunoprecipitate of eIF4GI682-1130-MS2 (**Figure 4C**). The eIF4GIe construct missing the core domain (eIF4GIeΔ682-1079-MS2) affected the reporter mRNA similar as the N-terminal portion of eIF4GIe alone (eIF4GIeNT-MS2): a moderate mRNA increase is observed with both reporter constructs A and B. The reason for this PTC proximity-independent effect is not known. As for eIF4GIeNT-MS2, expression of eIF4GIeΔ682-1079-MS2 was also lower than for the controls (**Figure 4D**, lower panel). Finally, the eIF4GIe construct comprising the C-terminal third of the protein (eIF4GI1080-1599-MS2) clearly had no effect on NMD, despite of its high expression level (**Figure 4D**).

Tethering of factors involved in translation to an mRNA could possibly influence the mRNA's translation rate, which would affect NMD since NMD is a translation-dependent mechanism. It was therefore important to test if tethering of PABPC1 or eIF4G altered translation of the tethered mRNA. To test this, we measured the translational activity with a luciferase reporter system (**Figure S3**). We used a *Renilla* luciferase (Rluc) construct containing 6 MS2 binding sites 82 nt downstream of the termination codon as a reporter (**Figure S3A**). As an internal reference und to normalize for variable transfection efficiencies, we co-transfected a firefly luciferase-encoding plasmid (Fluc). Both luciferase constructs were transfected into HeLa cells together with the indicated MS2 fusion protein-encoding plasmids and 48 hours post transfection the Rluc and Fluc activities in the cell lysates were measured. The relative light units (RLU) of Rluc were then divided by the RLU of Fluc (**Figure S3B**). These results showed that translation of the Rluc reporter was not significantly altered by tethering of full length PABPC1 (1–636-MS2), the first four RRMs of PABPC1 (1–372-MS2), eIF4GIe (eIF4GIe-MS2) or the eIF4GI core domain (eIF4GI682-1130-MS2) in comparison to tethering of the LacZ control (LacZ-MS2). Thus we conclude that the observed increase in NMD reporter mRNA caused by tethering of PABPC1 and eIF4G constructs is not simply a consequence of reduced translation of the reporter transcript.

Tethering eIF4GI and in particular also its core domain has been reported to direct translation re-initiation on a downstream ORF [54], [55] and re-initiation downstream of a PTC has been shown to inhibit NMD [56]. To address whether the observed suppression of NMD by tethered eIF4G might be due to re-initiation of translation downstream of the MS2 binding sites on our miniμ NMD reporter construct A, we generated a version of this reporter containing an in-frame C-terminal Flag tag (construct AF; **Figure S4A, B**) and tested it in tethering experiments. Paralleling the results obtained with construct A, RNA levels of reporter construct AF increased 4 to 5 times upon tethering of full length PABPC1 (1–636-MS2) or the eIF4GI core domain (eIF4GI642-1130-MS2; **Figure S4C**). Western blotting of the cell extracts from this experiment with an anti-Flag antibody did not reveal any signal indicative for translation of this downstream ORF (**Figure S4D**). The maximum mass of such a putative polypeptide, initiating immediately after the MS2 binding sites would be around 30 kDa, and initiation at an AUG about 240 nucleotides downstream of the MS2 binding sites would give rise to a 21 kDa polypeptide (**Figure S4B, A**). Re-initiation in a different frame cannot be excluded, but the two alternative frames are interrupted by numerous termination codons that again would be predicted to elicit NMD (**Figure S4A**). Thus, this result is consistent with the view that the eIF4G-mediated NMD suppression works by a mechanism different from re-initiation, although re-initiation formally cannot be ruled out.

Collectively, the results from tethering eIF4GI variants indicate that in addition to NMD inhibition through recruitment of PABPC1, eIF4GI can antagonize NMD by a second mechanism that involves the core domain of eIF4GI and is independent of PABPC1. Further our data suggest that these effects are not caused by interference of the tethered fusion proteins with translation.

### Tethering of CTIF does not inhibit NMD

It was reported that during early rounds of translation that can occur on cap binding complex (CBC)-bound mRNAs, the CBP80/20-dependent translation initiation factor (CTIF) functionally replaces eIF4GI [57]. Since PTC-containing CBC-bound mRNAs are targets for NMD [58]–[61] and since CTIF contains an MIF4G domain resembling the one in the core domain of eIF4GI (**Figure 4A**), we wanted to test if tethering of CTIF in the vicinity of the PTC on our NMD reporter transcript miniμ ter310 would also suppress NMD. Relative mRNA levels of reporter constructs A and B from cells expressing CTIF-MS2 were compared to those measured in cells expressing LacZ-MS2 (negative control) and PABPC1-MS2 (positive control, **Figure 4E**). Although well expressed, CTIF-MS2 did not increase miniμ ter310 reporter transcript, suggesting that with regards to NMD inhibition, CTIF cannot substitute for eIF4GI.

### eIF3 may be involved in inhibiting NMD through the eIF4GI core domain

Having revealed the capability of the tethered eIF4GI core domain to inhibit NMD independently of PABPC1, we next asked if this effect might be mediated by eukaryotic initiation factor 3 (eIF3). The central MIF4G domain of eIF4GI is well known to serve as the binding platform for eIF3 [62], [63], and previous studies showed that the eIF3 subunits eIF3f and eIF3h are involved in the protection of AUG-proximal PTCs from triggering NMD [14]. When either of these two factors were depleted by RNAi, otherwise NMD-resistant β-globin mRNAs with AUG-proximal PTCs became susceptible to NMD [14].

**Figure 5:**
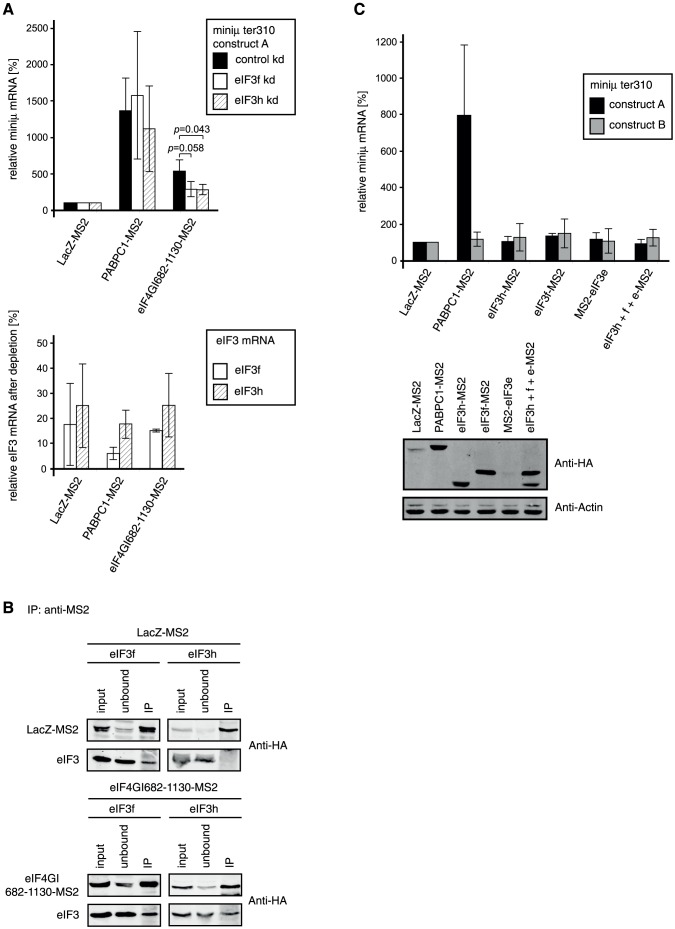
The NMD antagonizing function of the tethered eIF4GI core domain requires eIF3 subunits. (A) The tethering assay combined with depletion of eIF3f or eIF3h was performed as described in **Figure 3A**. The upper panel shows the relative miniμ ter310 reporter mRNA levels upon tethering of LacZ-MS2, PABPC1-MS2 or the eIF4GI core domain fused to MS2 (eIF4GI682-1130-MS2) in mock depleted cells (control kd) or cells depleted for eIF3f or eIf3h. Average values and standard deviations of three independent experiments are shown. The lower panel shows the relative mRNA levels of eIF3f and eIF3h in the respective knockdown samples, which were measured as a surrogate for protein levels to assess the knockdown efficacies, because no antibodies against these factors were available to us. Percentages of remaining eIF3f (white bars) or eIF3h (striped bars) mRNA relative to the corresponding control knockdown condition are shown. (B) Immunoprecipitations to probe for interactions between the eIF4GI core domain and eIF3 subunits f or h. HEK 293T cells were co-transfected with plasmids encoding LacZ-MS2 or eIF4GI682-1130-MS2 and eIF3f or eIF3h. After 48 h, immunoprecipitations were carried out with an anti-MS2 antibody and analyzed by western blotting using an anti-HA antibody (all proteins possess a C-terminal HA tag). The MS2 fusion proteins are shown in the upper panels, the eIF3 subunits in the lower panels of each immunoprecipitation. 10% of the cell extracts before (input) and the supernatant after (unbound) the immunoprecipitations, and 50% of the immunoprecipitated material (IP) were loaded on the gel. (C) Tethering assay as described in **Figure 1C**, but with different subunits of eIF3. LacZ-MS2 served as negative control and was set to 100%, PABPC1-MS2 served as positive control. The eIF3 subunits h, f and e were tested individually (eIF3f-MS2, eIF3h-MS2, eIF3e-MS2) and in combination with each other (eIF3f+h+e-MS2). Average values and standard deviations of three independent experiments are shown. The lower panel shows a western blot to monitor the expression levels of the MS2 fusion proteins using an anti-HA antibody. Endogenous β-actin was used to control for sample loading.

To test whether NMD inhibition induced by the tethered eIF4GI core domain (eIF4GI682-1130-MS2) requires the presence of eIF3f or eIF3h, we conducted the tethering assay in cells depleted for each of these factors (**Figure 5A**). To this end, a plasmid expressing an shRNA either against eIF3f or eIF3h was co-transfected along with plasmids encoding the eIF4GI-MS2 fusion protein and the miniμ ter310 reporter construct A. Due to the lack of specific antibodies, the efficacy of the eIF3f and eIF3h knockdowns was assessed by measuring the corresponding mRNA levels (**Figure 5A**, lower panel). As a control (control kd), an shRNA that does not target any known human mRNA was expressed. Depleting either of the two eIF3 subunits did not affect the capacity of PABPC1-MS2 to suppress NMD (**Figure 5A**, upper panel). However, the reporter mRNA increase induced by tethering of the eIF4GI core domain (eIF4GI682-1130-MS2) was reduced by approximately 40–50% when eIF3f or eIF3h was depleted as compared to the control knockdown. Although the effect is moderate, this result indicates that eIF3 is involved in the PABPC1-independent mechanism by which eIF4GI antagonizes NMD, but does not play a role in the PABPC1-dependent pathway. Notably, our result is reminiscent of the results obtained by Peixeiro and colleagues [14].

It should be noted that the reporter mRNA levels were much higher when PABPC1 was tethered than when the eIF4GI core domain was tethered (15-fold versus 6-fold). This is in contrast to the results of the standard tethering assays, where these two fusion proteins had a similar effect on the reporter (**Figure 4D**, compare PABPC1-MS2 and eIF4GI682-1130-MS2). One difference is that in the knockdown experiments, the time window for the expression of the fusion protein and the reporter transcript is extended. It is therefore possible that the reporter mRNA reaches its steady-state level earlier when the core domain is tethered than when PABPC1 is tethered.

*In vitro* experiments have previously shown that subunit e of eIF3 interacts directly with the eIF4GI core domain [64]. The same study however also suggests a close association of eIF3f and eIF3h (among other subunits) with eIF4GI. To test whether eIF3f and eIF3h actually interact with the eIF4GI682-1130-MS2 fusion protein, we co-expressed both factors transiently in HEK 293T cells, immunoprecipitated the eIF4G core domain via its MS2 fusion in presence of RNase A and tested for its association of eIF3f and eIF3h (**Figure 5B**). All recombinant proteins were HA-tagged for detection by western blot. Once more, LacZ-MS2 served as a specificity control for the immunoprecipitation. eIF3f and eIF3h both co-immunoprecipitated with eIF4GI682-1130-MS2, but while eIF3h was not detected in the control immunoprecipitation (LacZ-MS2), eIF3f even co-purified with LacZ-MS2. The apparently unspecific co-precipitation of eIF3f prevents us from drawing any conclusion regarding its association with the eIF4GI core domain. On the other hand, our data confirms an association between eIF3h and the core domain of eIF4GI.

Further evidence that the eIF4GI core domain interacts with eIF3 was revealed when we identified the proteins co-immunoprecipitating with eIF4GI682-1130-MS2 by mass spectrometry (**Table S1**). Among the 22 top scoring interactors all 13 eIF3 subunits were detected, strongly suggesting that eIF4GI associated with the entire eIF3 complex.

Finally, we asked if the two eIF3 subunits f and h had the capacity to stabilize a NMD reporter transcript in a tethering experiment (**Figure 5C**). In addition, we also tested eIF3e, which directly interacts with eIF4GI [64] and which has also been implicated in NMD albeit as an NMD promoting rather than inhibiting factor [65]. The relative mRNA levels of the two miniμ ter310 constructs A and B were measured by RT-qPCR after co-expression with eIF3h-MS2, eIF3f-MS2, eIF3e-MS2, or all three eIF3 subunits together (eIF3h+f+e-MS2). LacZ-MS2 and full length PABPC1-MS2 served as controls. As is readily visible in **Figure 5C** (upper panel), none of these eIF3 subunits was able to inhibit NMD when tethered into the vicinity of a PTC, neither individually nor in combination. In contrast to subunits h and f that expressed at high levels in these experiments, it should be noted that eIF3e expression consistently remained very low (**Figure 5C**, lower panel).

Taken together, the reduced capacity of the tethered eIF4GI core domain to suppress NMD in the eIF3f and eIF3h knockdowns (**Figure 5A**) and the association between the eIF4GI core domain and eIF3 (**Figure 5B** and **Table S1**) identify the eIF4G-eIF3 connection as part of a new NMD antagonizing pathway that is genetically separable from the previously described PABPC1-mediated NMD inhibition. That tethering of individual eIF3 subunits failed to inhibit NMD (**Figure 5C**) could simply be due to the inability of these MS2 fusion proteins to assemble functional eIF3 complexes.

## Discussion

Following up on our own previous study [22] and the work of several other labs [15], [21], [23]–[25] showing that PABP antagonizes NMD, we identified here the first two RRMs of PABPC1 as necessary and the first three RRMs as sufficient for suppressing NMD in a tethering assay (**Figure 1**). The linker domain clearly also contributed to the NMD suppressing function of PABPC1, likely by its capacity to multimerize PABPC1 to the reporter transcript. Surprisingly and contradictory to previously reported data [24], the eRF3 interacting C-terminal PABC domain of PABPC1 was dispensable for suppression of NMD in our hands. Therefore, our results do not support the model that NMD simply depends on a competition between UPF1 and PABPC1 for binding to eRF3 [25], [31]. Similar to our results and pointing to a crucial and conserved function of the RRMs, tethering of RRM1-4 of the yeast Pab1p also led to a robust inhibition of NMD, albeit weaker than full length Pab1p, whereas tethering of the C-terminal half of Pab1p barely stabilized the NMD reporter transcript [15]. The finding that a fragment of Sup35p (the yeast eRF3 ortholog) lacking the Pab1p interacting domain could suppress the slow growth phenotype of a *sup35Δ* strain and was able to stabilize the NMD reporter when tethered downstream of the PTC further implies that the PABP:eRF3 interaction is not essential for normal translation termination and for antagonizing NMD [32].

Since the portion of PABPC1 that is necessary and sufficient for suppressing NMD encompasses the binding to eIF4G, we tested if the PABPC1:eIF4G interaction was critical for stabilization of the NMD reporter mRNA (**Figure 2**). Consistent with this idea, the M161A point mutation reduced PABPC1's capacity to inhibit NMD (**Figures 2A** **and S2**). The fact that the RRM1-4 PABPC1 construct carrying the M161A mutation still co-precipitated to some extent with eIF4GI-MS2 (**Figure 2B**) indicates that this originally *in vitro* identified and tested point mutation [41], [52] is not sufficient to completely prevent binding to eIF4G *in vivo*.

Further evidence for a critical role of the PABPC1:eIF4G interaction for the suppression of NMD was provided by PABPC1 tethering experiments in cells depleted for eIF4G, which resulted in diminished reporter mRNA levels (**Figure 3**). Since this interaction is required for the PABP:eIF4G:eIF4E-mediated circularization of mRNAs, our results are consistent with the idea that this “closed-loop” mRNP structure is critical for correct translation termination and recycling of ribosomal subunits.

This hypothesis predicted that tethering of eIF4G should also suppress NMD. Consistent with this view, the full-length eIF4GI isoforms capable of interacting with PABPC1 (isoforms d, e and f) increased the reporter transcript in tethering assays (**Figure 4B, C**). A moderate increase was also induced by tethering the eIF4GIe constructs NT and Δ682-1079, which both contain the binding sites for PABPC1 and for eIF4E and hence could potentially bring the 5′ and the 3′ end of the reporter mRNA close to the PTC (**Figure 4D**). However, complicating the situation, these two eIF4GI variants had essentially the same effect on the construct B reporter mRNA, indicating that the observed reporter mRNA increase in this case did not require the PABP:eIF4G:eIF4E complex to be close to the PTC. It is therefore also not clear, whether this mRNA increase resulted from an inhibition of NMD or from a general mRNA stabilization.

Most interestingly, tethering of the eIF4G core domain alone, encompassing the RRM and the MIF4G domain, also efficiently suppressed NMD. Since the eIF4G core domain lacks both the PABPC1 and the eIF4E binding sites, this effect cannot be attributed to formation of a “closed loop” configuration and therefore provides evidence for an independent second mechanism of NMD suppression. We hypothesized that eIF4G in the vicinity of a PTC might inhibit NMD by promoting re-initiation of translation further downstream on the reporter mRNA. However, our attempt to detect polypeptides originating from such putative re-initiation events on the miniμ reporter transcript failed (**Figure S4**). Thus, although we have no evidence for re-initiation being involved, we cannot either rule it out based on these negative results.

Finding that the eIF4G core domain was capable of antagonizing NMD suggested that the same might be true for CTIF, because CTIF contains a highly homologous MIF4G domain and was reported to functionally replace eIF4G during translation initiation of CBC-associated mRNAs [57]. In the tethering assay, however, CTIF was not capable of antagonizing NMD despite of its robust expression (**Figure 4E**). The specific motifs in the eIF4G core domain responsible for the observed NMD suppression remain therefore to be identified.

A well-characterized interactor of the eIF4G core domain is the eIF3 complex [62], [63]. Besides its function in translation initiation, eIF3 was shown to be involved in disassembling the post-termination ribosome and recycling of the ribosomal subunits in a reconstituted *in vitro* system [66]. Moreover, a role for eIF3 in translation termination has recently been documented in yeast cells [67]. Specifically, the pulldown assays of Beznoskova and colleagues provided evidence for an association of eIF3 with release factors Sup45p (eRF1 ortholog) and Sup35p (eRF3 ortholog) as well as with the ribosome recycling factor Rli1 (ABCE1 ortholog). Furthermore, there is also evidence for a link between eIF3 and NMD, but collectively the data does not provide an easily interpretable picture: subunit a was shown to interact with phosphorylated UPF1 [68], subunit e was identified as an essential NMD factor associated with UPF2 [65] and the CTIF-interacting subunit g inhibits NMD when down-regulated [69], whereas subunits f and h are required to prevent NMD of β-globin reporter transcripts with AUG proximal PTCs [14].

Knockdown of eIF3f and h rendered the otherwise NMD-resistant β-globin transcript with a PTC at codon 15 sensitive to NMD [14]. Peixeiro and colleagues speculated that eIF3 remains associated with the ribosome during the early phase of translation, thereby retaining the ribosome's capacity to terminate correctly and hence inhibit NMD. Our finding that the eIF4GI core domain-mediated NMD inhibition is sensitive to decreased eIF3f and h levels (**Figure 5A**) is consistent with the proposed function of these two eIF3 subunits as promoters for correct translation termination. However, direct tethering of eIF3f and h, individually or together, to our NMD reporter construct had no effect on the reporter mRNA level (**Figure 5C**). Such a negative result in a tethering assay could always be due to technical issues, for example functional inactivation of a protein by its fusion to the MS2 coat protein, and remains therefore inconclusive. Nevertheless, our data collectively corroborate previous reports indicating a key role of eIF3 in the process of translation termination and therewith in the decision of whether or not NMD gets triggered.

In summary, the work presented here delineates two apparently independent pathways involved in preventing NMD. One relies on eIF4G interacting with PABP and hence presumably on the formation of the “closed-loop” mRNP structure, and the other involves the eIF4G-binding eIF3 complex. More research is necessary to elucidate the precise function of the eIF3 complex in translation termination and NMD and the consequence of the closed-loop mRNP structure for these processes.

## Materials and Methods

### Plasmids

The MS2 binding sites-containing miniμ and TCRβ reporter genes were described previously [22]. Miniμ ter310 construct AF was generated by adding a Flag tag sequence to the 3′end of the miniμ ter310 construct A reporter using fusion PCR. For the tethering experiments, the different mutants/deletions of PABPC1 were generated by PCR or fusion-PCR with pCMV-PABPC1-MS2-HA [22] as a template (for plasmids and primer sequences see **Table S2** and **Table S3**). The PCR-amplified fragments were cut with KpnI and BamHI and cloned into a KpnI-BamHI pCMV-MS2-HA vector backbone. Site-directed mutagenesis to generate the M161A mutation in PABPC1 was performed using the QuikChange XL Site-Directed Mutagenesis Kit (Stratagene) using oligonucleotides 5′- GCTATTGAAAAAATGAATGGAGCGCTCCTAAATGATCG-3′ and 5′- CGATCATTTAGGAGCGCTCCATTCATTTTTTCAATAGC-3′ with the mutation. PABPC1 variants without the MS2 fusion protein were created by PCR amplification and insertion into a pCMV-HA containing vector backbone using NheI and SacII restriction sites.

For the eIF4GI mutants/deletions, the N-terminal myc-tag in pCDNA3.1-myc-eIF4GIf (obtained from Simon Morley, University of Sussex) [70] was replaced by a PCR amplified DNA fragment containing HA-MS2, generating pCDNA3.1-HA-MS2-eIF4GIf (not used in this study). This plasmid was subsequently used for the generation of pCMV-eIF4GIf-MS2-HA by PCR amplification of the eIF4GIf ORF and insertion into pCMV-PABPC1-MS2-HA with PvuI and KspI. The MS2-HA sequence had to be reintroduced by PCR amplification of the sequence using pCMV-PABPC1-MS2-HA as template and insertion in between the KspI and XbaI restriction sites (primers rj26 and om214, **Table S3**). eIF4GI isoforms were PCR-amplified using pCMV-eIF4GIf-MS2-HA as template and inserted between the PvuI and SpeI restriction sites of the same vector. The rest of the eIF4GI constructs were generated by PCR or in the case of pCMV-eIF4GIeΔ682-1079-MS2-HA by fusion PCR using pCMV-eIF4GIf-MS2-HA as template. They were inserted into pCMV-eIF4GIf-MS2-HA between the PvuI and SacII restriction sites. pCMV-eIF3f-MS2-HA and pCMV-eIF3h-MS2-HA were PCR amplified from HeLa cDNA and inserted into pCMV-eIF4GIf-MS2-HA using the PvuI and SacII restriction sites. pCMV-HA-MS2-eIF3e was created by PCR amplification of eIF3e using a template plasmid obtained from John W. Hershey (UC Davis Cancer Center, Sacramento, CA) and inserting it into a pCMV-HA-MS2 vector backbone using the HindIII and XhoI restriction sites. MS2-less eIF3 vectors were created by cloning the eIF3 open reading frame into a pCMV-HA containing vector backbone using PvuI and SacII restriction sites. pCMV-CTIF-MS2-HA was created by PCR amplification of the CTIF ORF using pCDNA3-FLAG-CTIF as template (obtained from Yoon Ki Kim, Korea University, Seoul) [57]. The amplified fragment was inserted into pCMV-PABPC1-MS2-HA using the BamHI and SalI restriction sites. pCMV-MS2-HA and pCMV-LacZ-MS2-HA were described previously [22]. pCMV-SLBP-MS2-HA was created by PCR amplification of the SLBP ORF using pcDNA3-HA-SLBP (obtained from Daniel Schümperli, University of Bern) as template and inserting it into pCMV-PABPC1-MS2-HA using the KpnI and BamHI restriction sites. The pCMVrGPx1-TGC vector used for normalization was described previously [71]. pcDNA3-HA-EGFP used for normalization in the depletion experiments was provided by Daniel Schümperli [72].

For the knockdowns, oligonucleotides encoding for shRNAs were inserted between the BglII and HindIII restriction sites in the pSUPERpuro vector [73]. The target sequence for depletion of eIF4GI was 5′-GAGCGAAGCTGCTGCAGAA-3′ [74]. For eIF3f it was 5′-ATACGCGTACTACGACACT-3′ and for eIF3h 5′-GATCGGCTTGAAATTACCA-3′ [14]. The sequence for the control shRNA is described elsewhere [75]. The inserts of all plasmids were verified by Sanger sequencing. Sequences are available upon request.

For the luciferase assay experiment, a pcDNA vector containing the *Renilla* luciferase ORF with 6 MS2 binding sites 82 nt downstream of the termination codon was used (pcDNA-Renilla-6MS2bs, obtained as a gift from Melissa J. Moore). As a normalizer, a pCI-neo plasmid (Promega) with the firefly luciferase ORF inserted between the NheI and NotI restriction sites (pCI-neo-FL) was used (a kind gift from Christoph Schweingruber).

### Cell culture

HeLa and HEK 293T cells were grown in Dulbecco's modified Eagle's medium (DMEM, Invitrogen) supplemented with 10% fetal calf serum (FCS), 100 U/mL penicillin and 100 µg/mL streptomycin under a 5% CO_2_ atmosphere.

### Transient transfection and RT-qPCR

For the tethering experiments 2×10^5^ HeLa cells per sample were seeded into 6-well plates. The next day transfection was performed using DreamFect (OZ Biosciences) transfection reagent with 100 ng NMD reporter (miniμ ter310 construct A or B; or TCRβ ter68 construct A or B), 50–100 ng pCMVrGPx1-TGC [71] as a normalizer and 400–800 ng of the MS2 fusion protein per sample. 400 ng were used for CTIF, PABPC1 and eIF3 variants, 800 ng were used for LacZ and eIF4GI variants. The cells were harvested 48 h after transfection. For the tethering experiments in **Figure 1** and **Figure S1**, total RNA was extracted with the “Absolutely RNA RT-PCR Miniprep Kit” (Stratagene). For the rest of the experiments, total RNA was extracted using the Guanidium Thiocyanate–Phenol–Chloroform Extraction as described previously [76]. Reverse transcription was performed with 1 µg of RNA in a volume of 20 µL using 300 ng random hexamers, 0.4 nM dNTPs, 10 mM DTT, 1× AffinityScript RT Buffer and 1 µL of AffinityScript Multiple Temperature Reverse Transcriptase (Agilent Technologies) according to the manufacturer's manual. qPCR measurements for experiments in **Figure 1** and **Figure S1** was performed as described earlier [75]. The other experiments were measured with the same mRNA assays, but with Brilliant III Ultra Fast QPCR Master Mix (Agilent Technologies) using a Rotorgene 6000 (Corbett). eIF3f and eIF3h mRNA in **Figure 5A** was measured using Brilliant III Ultra Fast SYBR Green QPCR Master Mix (Agilent Technologies), primers 5′-CACCCAGTCATTTTGGCCTC-3′ and 5′-CGACAGTTCCCAACAGGGTC-3′ (5 µM each) for eIF3f and primers 5′-CTGCTCATTGCAGGCCAGAT-3′ and 5′-GAGCCTGGGCCATGAAGAG-3′ (5 µM each) for eIF3h [14].

### RNAi

Knockdowns were achieved by transfecting pSUPERpuro plasmids containing appropriate target sequences. Transfections were carried out as described above, except that 400 ng pSUPERpuro plasmid was added to the transfections. 18–20 h after transfection, 1.5 µg/mL puromycin was added to the cell culture medium. After 48 h under selection, the cells were cultured for the final 24 h in puromycin-free medium. 96 h post transfection, the cells were harvested.

### Co-immunoprecipitation

3×10^6^ HEK 293T cells were seeded in 10-cm dishes and transfected the next day with polyethyleneimine (105 mM, pH 7). 0.4 µL/µg DNA were used. For PABPC1 and eIF3 constructs 3 µg plasmid, for LacZ and eIF4GI constructs 10 µg plasmid was used. After 48 hours, 6×10^6^ cells were harvested, washed in PBS and lysed for 10 minutes in hypotonic gentle lysis buffer (10 mM Tris-Hcl pH 7.5, 10 mM NaCl, 2 mM EDTA, 0,5% Triton X-100 supplemented with 1× Halt Protease Inhibitor from Thermo Scientific and 125 µg/mL RNase A) on ice. After 10 min NaCl was brought to 150 mM. After centrifugation (16000 g, 15 min, 4°C), 200 µL supernatant was removed and stored as “input”. The rest (400 µl) was incubated with 5 µg Anti-Enterobacterio Phage MS2 Coat Protein antibody (Millipore) for 2 h at 4°C on a turning wheel. 30 µL Dynabeads Protein G (Life Technologies) were then added and incubated for 2 h at 4°C on a turning wheel. 200 µL was afterwards stored as “unbound”. The samples were then washed five times with 1 ml 10 mM Tris-HCl (pH 8), 150 mM NaCl, 0.1% NP-40. Immunoprecipitates were stored in 100 µL 2× SDS buffer (2% SDS, 60 mM Tris pH 6.6, 10% glycerol, 200 mM DTT) and separated by SDS-PAGE followed by western blotting.

### Immunoblotting

For the tethering experiments total cell extract was gained from aliquots of the harvested cells. Hypotonic gentle lysis buffer (10 mM Tris-Hcl pH 7.5, 10 mM NaCl, 2 mM EDTA, 0,5% Triton X-100 supplemented with 1× Halt Protease Inhibitor from Thermo Scientific) was added to a concentration of 10000 cells/µL and incubated on ice for 10 min. After centrifugation (16000 g, 15 min, 4°C) the same amount of 2× SDS buffer (2% SDS, 60 mM Tris pH 6.6, 10% glycerol, 200 mM DTT) was added (final concentration 5000 cells/µL).

For the tethering experiments an equivalent of 2×10^5^ cells were separated by electrophoresis on a 10% (PABPC1 and eIF3 fusion proteins) or a 7% (eIF4GI fusion proteins) SDS-polyacrylamide gel. For the immunoprecipitations an equivalent of 4×10^5^ cells were loaded for “input” and “unbound”, and the equivalent of 2×10^6^ cells were loaded for “IP”. The proteins were transferred onto Optitran BA-S 85 reinforced nitrocellulose (GE Healthcare) by electro-blotting. Primary antibodies and dilutions used were Mouse anti-HA C5 (Enogene Biotech, **Figures 4D, 4E** **,** **5C** **, S3**) 1∶1000, mouse anti-HA 12CA5 (Roche, Figures 1C, 2B, S3) 1∶1000, rabbit anti-HA Y11 (Santa Cruz, **Figures 2A** **,** **3B** **,** **4B, 4C** **,** **5B** **, S1**) 1∶1000, mouse anti-Flag OctA probe (D-8) (Santa Cruz, Figure S4C), 1∶1000, mouse anti-SMB/B′ [77] (**Figure 1C**) 1∶400, rabbit anti-actin (Sigma Aldrich, **Figures 5C** **, S3**) 1∶1000, mouse anti-tubulin B-7 (Santa Cruz, **Figures 2A** **,** **3B** **,** **4B**) 1∶1000, mouse anti-Tyrosine-Tubuline (Sigma Aldrich, **Figure 4D, 4E**) 1∶5000, rabbit anti-CPSF100 [78] (**Figure S2**) 1∶10000, rabbit anti CPSF73 [79] (**Figure S3**) 1∶5000, rabbit anti-eIF4GI (obtained from Nahum Sonenberg, McGill University Montreal; **Figure 3B**) 1∶1000, mouse anti-PABPC1 10E10 (Santa Cruz, **Figures 4C** **, S1**) 1∶1000. Secondary antibodies from LI-COR Biosciences used were donkey anti-rabbit IRDye800CW (**Figure 2A**), donkey anti-mouse IRDye680LT (**Figure 2A**), goat anti-mouse IRDye800CW (**Figures 2B** **,** **4C, 4D, 4E** **,** **5C** **, S1**), goat anti-rabbit IRDye800CW (**Figures 3A** **,** **4B, 4C** **,** **5B, 5C**) and goat anti-mouse IRDye680LT (**Figures 3A** **,** **4B** **, S1**), all diluted 1∶10000. An Odyssey Infrared Imaging System was used for detection (LI-COR Biosciences). For **Figures 1C** **and S3**, HRP-conjugated anti-rabbit IgG and HRP-conjugated anti-mouse IgG (Promega, diluted 1∶2500) were used as secondary antibodies and chemiluminescence was detected using ECL+ Plus Western blotting detection system (Amersham) and a Luminescent Image Analyzer LAS-1000 (Fujifilm).

### Luciferase assay

For the luciferase assay experiments, two wells with 2×10^5^ HeLa cells per sample were seeded into 6-well plates. The next day 100 ng Renilla luciferase reporter, 20 ng Firefly luciferase reporter and 400 ng MS2 fusion protein per well were transiently transfected using DreamFect (OZ Biosciences) transfection reagent. One day later the cells from each sample were pooled and redistributed equally into 3 wells on a 6-well plate. The cells were harvested 48 h after transfection according to the Dual-Luciferase Reporter Assay System (Promega) technical manual. The luciferase measurements were subsequently performed according to the instructions in the technical manual on a Tecan Infinite M1000PRO microplate reader.

### Mass spectrometry

Samples were reduced with 50 mM DTT in 50 mM Tris/HCl pH 8 at 50°C for 30 min, and alkylated at 37°C for 30 min in the dark with 50 mM IAA Solution in 50 mM Tris/HCl pH 8, and digested 1 h at 50°C with Trypsin 100 ng/µL. Each digest was analyzed in LCMS. A volume of 10 µL was loaded onto a self-made pre-column (Magic C18, 5 mm, 300 Å, 0.15 mm i.d.×30 mm length) at a flow rate of ∼5 ml/min with solvent A (0.1% formic acid in water/acetonitrile 98:2). After loading, peptides were eluted in back flush mode onto the analytical nano-column (Magic C18, 5 mm, 100 Å, 0.075 mm i.d. ×75 mm length) using an acetonitrile gradient of 5% to 40% solvent B (0.1% formic acid in water/acetonitrile 4.9:95) in 40 min at a flow rate of ∼400 nl/min. The column effluent was directly coupled to an LTQ-orbitrap XL mass spectrometer (ThermoFisher, Bremen, Germany) via a nanospray ESI source operated at 1700 V. Data acquisition was made in data dependent mode with precursor ion scans recorded in the Fourier transform detector (FT) with resolution of 60'000 (@ m/z = 400) parallel to five fragment spectra of the most intense precursor ions in the linear iontrap. CID mode settings were: Wideband activation on; precursor ion selection between m/z range 360–1400; intensity threshold at 500; precursors excluded for 15 sec. CID spectra interpretation was performed with EASYPROT on a local, dual quad core processor server run under linux. The following variable modifications were used: carboamidomethylated Cys (no limit), Met oxidation (limited to 1), Asn/Gln deamidation (2), phosphorylation (limited to 1). Parent and fragment mass tolerances were set to 10 ppm and 0.6 Da, respectively. All protein identifications were accepted with a false discovery rate of 1% and a number of unique peptides superior or equal to 2. Semi-quantitative protein abundance is assessed with the PMSS score, which is the sum of the z score of peptides belonging to the same protein

## Supporting Information

Figure S1 **LacZ-MS2 is a better control than MS2 alone.** HeLa cells were transiently transfected with the TCRβ ter68 reporter construct A or construct B together with an MS2 fusion protein and GPx1 as a normalizer. The MS2 protein alone (MS2), the truncated LacZ with a C-terminal MS2 fusion (LacZ-MS2) and the histone RNA hairpin-binding protein with a C-terminal MS2 fusion (SLBP-MS2) were tested. Additionally a plasmid not containing any MS2-fusion protein was used (Mock). After 48 hours total RNA was extracted followed by RT-qPCR. Reporter mRNA increase was calculated in relation to the control (LacZ-MS2, set to 100). The result of one experiment is shown.
(PDF)

Figure S2 **The M161A mutation also reduces the stabilization of a TCRβ ter68 NMD reporter.** Tethering assay with a TCRβ reporter construct. Construct A has a cassette of 6 MS2 binding sites directly after the PTC at amino acid position 68, construct B has the same cassette further downstream [22]. HeLa cells were transiently transfected with the NMD reporter TCRβ ter68 construct A or construct B and plasmids encoding the indicated MS2-fusion proteins (LacZ, 1–636, 1–372, 1–372M161A). GPx1 was again used as a normalizer. Each bar represents the average and standard deviation of six independent experiments. On the right a western blot is shown using an anti-HA antibody to detect the transfected MS2-fusion proteins and antibodies against CPSF100 and actin as loading controls.
(PDF)

Figure S3 **Tethering of PABPC1 and eIF4GI MS2 fusion proteins to a reporter mRNA does not influence its translational activity.** (A) Schematic representation of the Renilla luciferase reporter (Rluc 6MS2). (B) Renilla luciferase activity was measured upon tethering of various MS2-fusion proteins. HeLa cells were transiently transfected with a plasmid vector containing the Renilla luciferase ORF followed by 6 MS2 binding sites 82 nt downstream of the translation termination codon and plasmids encoding the indicated MS2 fusion proteins (LacZ, 1–636, 1–372, eIF4GIe, eIF4GI682-1130). A Firefly luciferase containing plasmid vectors was cotransfected as a normalizer. The results are shown as the ratio between the measured Relative Light Units (RLU) of both luciferases (RLU RLuc/FLuc). Each bar represents the average and standard deviation of three independent experiments. On the right a western blot is shown using an anti-HA antibody to detect the transfected MS2-fusion proteins. An antibody against CPSF73 was used as loading control. Due to the size of eIF4GIe, the corresponding sample had to be run on a different gel than the rest of the samples (eIF4GIe-MS2).
(PDF)

Figure S4 **No evidence for translation reinitiation downstream of the MS2 binding sites.** (A) Amino acid sequences encoded in all 3 frames by the miniμ sequence downstream of the MS2 binding sites in reporter construct A. To monitor putative translation of the single longer ORF (in frame 2), a Flag-tag was inserted immediately before the stop codon into miniμ ter310 construct A giving construct AF (schematically illustrated in (B)). The longest possible ORF, initiating at a non-AUG directly 3′ of the MS2 binding sites (6MS2) and terminating after the Flag tag, would result in a polypeptide with a molecular weight of ∼30 kDa (dashed box, max. ∼30 kDa). Reinitiation at the indicated AUG in frame 2 would generate a ∼21 kDa polypeptide. (C) Testing if tethered PABP or eIF4G promotes translation reinitiation. HeLa cells were transiently transfected with miniμ ter310 constructs A or AF and plasmids encoding the indicated MS2-fusion proteins (LacZ, PABPC1 1–636, eIF4GI 642–1130). Cotransfected GPx1 was used as a normalizer. (D) Western blot showing expression of the MS2 fusion proteins using an anti-HA antibody (upper panel). In the central panel an anti-Flag antibody was used. As a control HeLa cell extract containing a transiently transfected blue fluorescent protein with a C-terminal Flag tag was loaded (EBFP-Flag). Actin was used as a loading control (lower panel). The amount of cell equivalents loaded is indicated at the top of the corresponding lane.
(PDF)

Table S1 **Proteins identified to interact with the core domain of eIF4GI.** Mass spectrometric analysis was carried out on immunoprecipitations of eIF4GI682-1130-MS2. The identified proteins are ordered by Protein Match Score Summation (PMSS).
(PDF)

Table S2 **Plasmids used in this study.** The full name, the oligonucleotides used for cloning and a short description are indicated. The oligonucleotide sequences can be found in Table S3.
(PDF)

Table S3 **Oligonucleotides used for cloning in this study.**
(PDF)
