# Supplementary figures and images for: Eukaryotic Initiation Factor 4G Suppresses Nonsense-Mediated mRNA Decay by Two Genetically Separable Mechanisms
Source: PLoS One. 2014 Aug 22;9(8):e104391. doi: 10.1371/journal.pone.0104391 (PMC4141738; doi:10.1371/journal.pone.0104391)

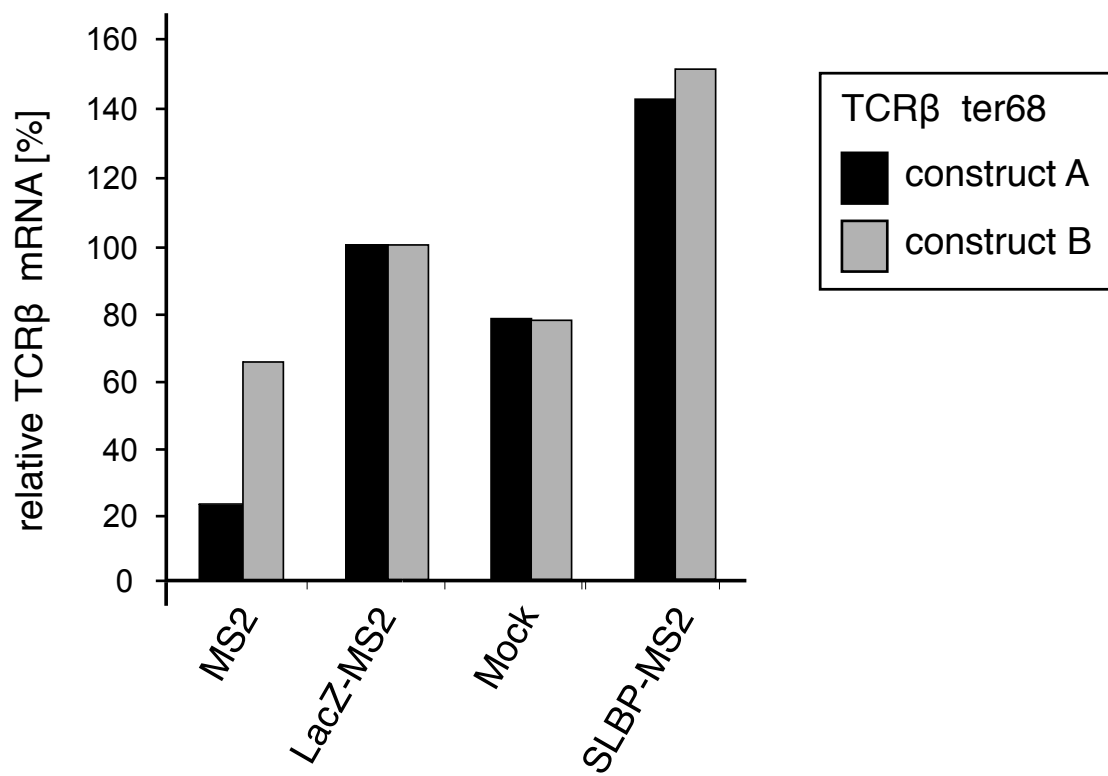

Supplement: Figure S1 — LacZ-MS2 is a better control than MS2 alone. HeLa cells were transiently transfected with the TCRβ ter68 reporter construct A or construct B together with an MS2 fusion protein and GPx1 as a normalizer. The MS2 protein alone (MS2), the truncated LacZ with a C-terminal MS2 fusion (LacZ-MS2) and the histone RNA hairpin-binding protein with a C-terminal MS2 fusion (SLBP-MS2) were tested. Additionally a plasmid not containing any MS2-fusion protein was used (Mock). After 48 hours total RNA was extracted followed by RT-qPCR. Reporter mRNA increase was calculated in relation to the control (LacZ-MS2, set to 100). The result of one experiment is shown. (PDF) [file pone.0104391.s001.pdf]

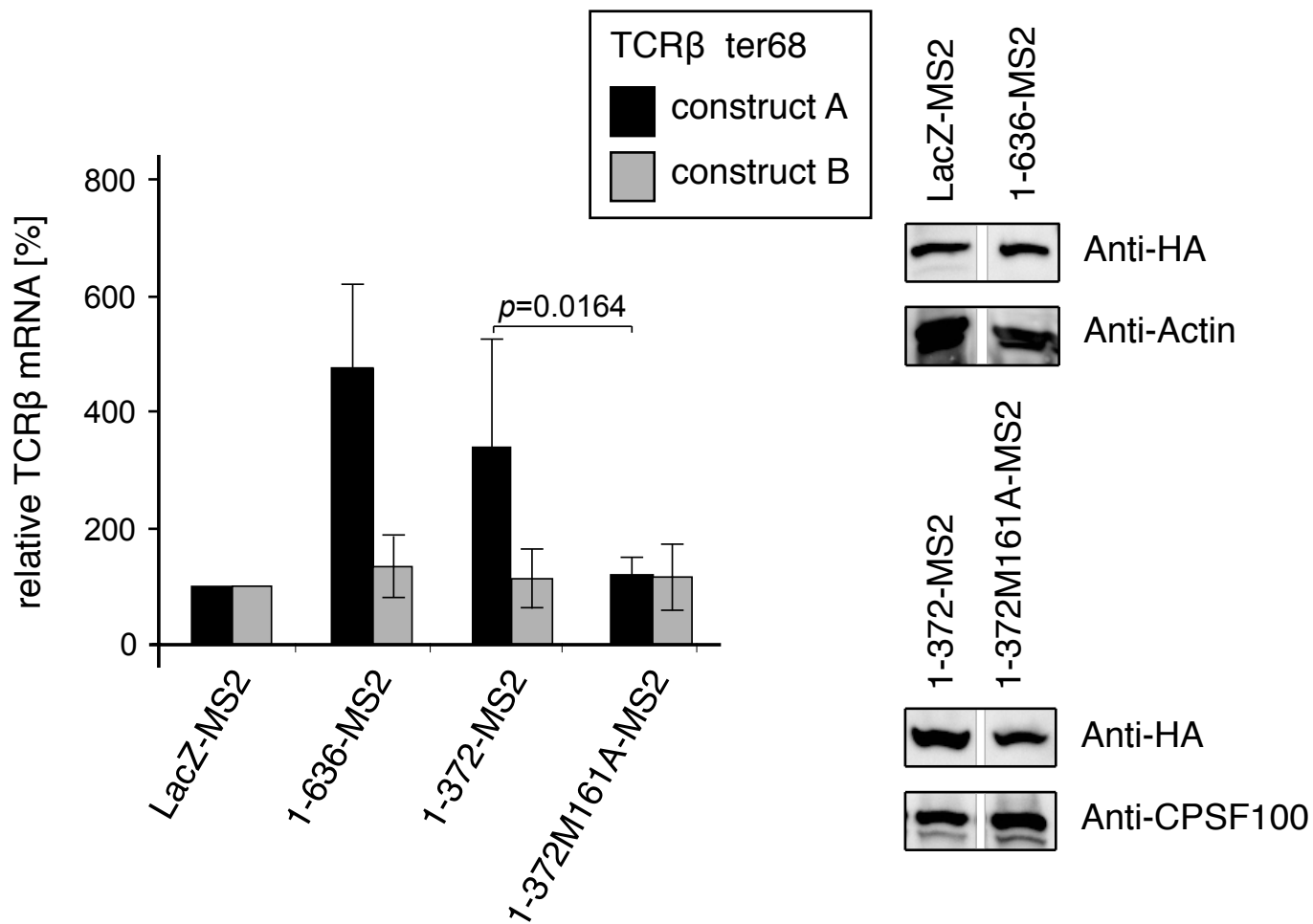

Supplement: Figure S2 — The M161A mutation also reduces the stabilization of a TCRβ ter68 NMD reporter. Tethering assay with a TCRβ reporter construct. Construct A has a cassette of 6 MS2 binding sites directly after the PTC at amino acid position 68, construct B has the same cassette further downstream [22]. HeLa cells were transiently transfected with the NMD reporter TCRβ ter68 construct A or construct B and plasmids encoding the indicated MS2-fusion proteins (LacZ, 1–636, 1–372, 1–372M161A). GPx1 was again used as a normalizer. Each bar represents the average and standard deviation of six independent experiments. On the right a western blot is shown using an anti-HA antibody to detect the transfected MS2-fusion proteins and antibodies against CPSF100 and actin as loading controls. (PDF) [file pone.0104391.s002.pdf]

**A**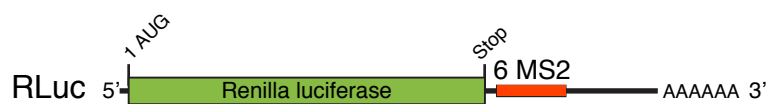**B**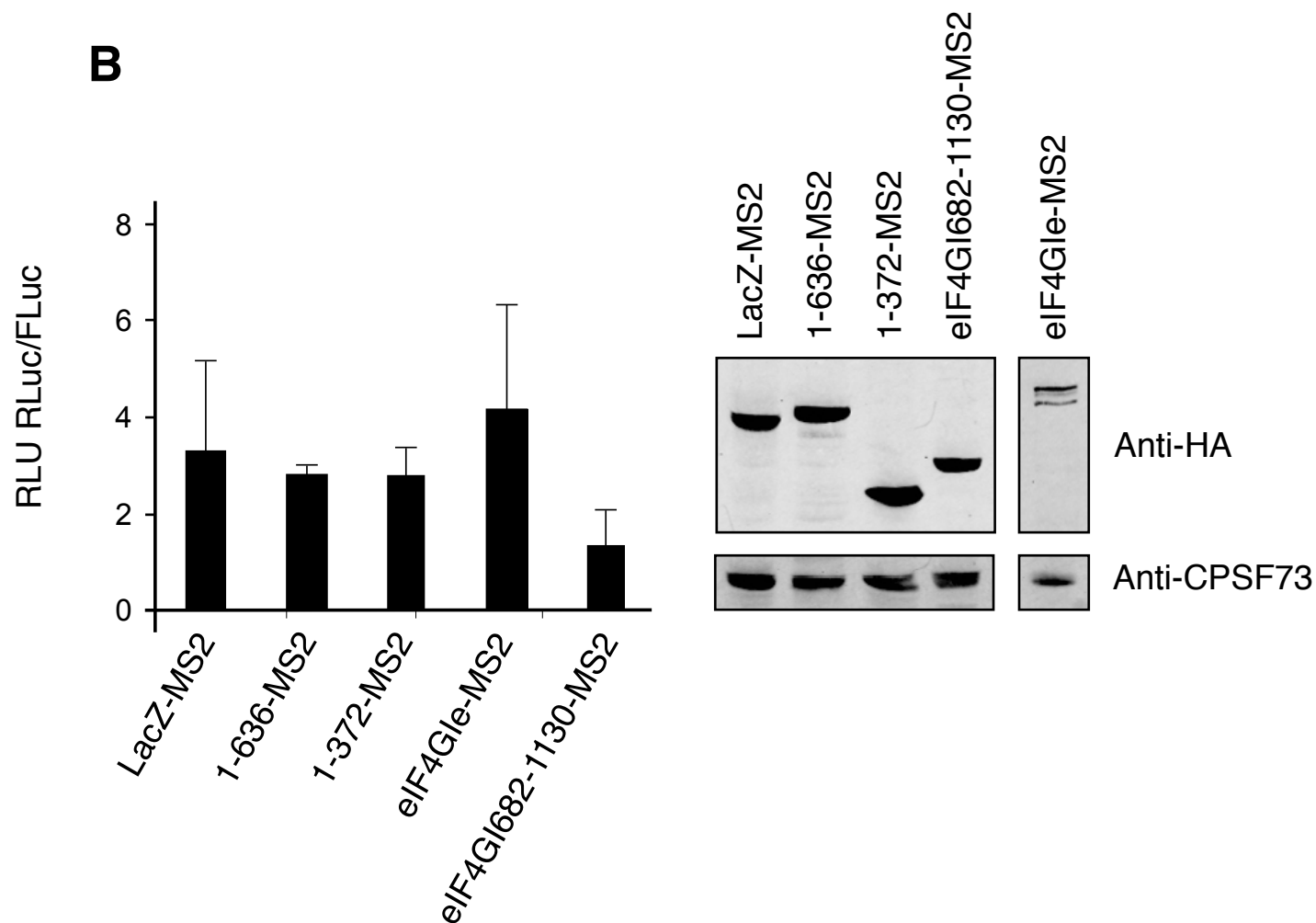

Supplement: Figure S3 — Tethering of PABPC1 and eIF4GI MS2 fusion proteins to a reporter mRNA does not influence its translational activity. (A) Schematic representation of the Renilla luciferase reporter (Rluc 6MS2). (B) Renilla luciferase activity was measured upon tethering of various MS2-fusion proteins. HeLa cells were transiently transfected with a plasmid vector containing the Renilla luciferase ORF followed by 6 MS2 binding sites 82 nt downstream of the translation termination codon and plasmids encoding the indicated MS2 fusion proteins (LacZ, 1–636, 1–372, eIF4GIe, eIF4GI682-1130). A Firefly luciferase containing plasmid vectors was cotransfected as a normalizer. The results are shown as the ratio between the measured Relative Light Units (RLU) of both luciferases (RLU RLuc/FLuc). Each bar represents the average and standard deviation of three independent experiments. On the right a western blot is shown using an anti-HA antibody to detect the transfected MS2-fusion proteins. An antibody against CPSF73 was used as loading control. Due to the size of eIF4GIe, the corresponding sample had to be run on a different gel than the rest of the samples (eIF4GIe-MS2). (PDF) [file pone.0104391.s003.pdf]
