# Supplementary material for: Eukaryotic Initiation Factor 4G Suppresses Nonsense-Mediated mRNA Decay by Two Genetically Separable Mechanisms
Source: PLoS One. 2014 Aug 22;9(8):e104391. doi: 10.1371/journal.pone.0104391 (PMC4141738; doi:10.1371/journal.pone.0104391)
Supplement: Figure S4 — No evidence for translation reinitiation downstream of the MS2 binding sites. (A) Amino acid sequences encoded in all 3 frames by the miniμ sequence downstream of the MS2 binding sites in reporter construct A. To monitor putative translation of the single longer ORF (in frame 2), a Flag-tag was inserted immediately before the stop codon into miniμ ter310 construct A giving construct AF (schematically illustrated in (B)). The longest possible ORF, initiating at a non-AUG directly 3′ of the MS2 binding sites (6MS2) and terminating after the Flag tag, would result in a polypeptide with a molecular weight of ∼30 kDa (dashed box, max. ∼30 kDa). Reinitiation at the indicated AUG in frame 2 would generate a ∼21 kDa polypeptide. (C) Testing if tethered PABP or eIF4G promotes translation reinitiation. HeLa cells were transiently transfected with miniμ ter310 constructs A or AF and plasmids encoding the indicated MS2-fusion proteins (LacZ, PABPC1 1–636, eIF4GI 642–1130). Cotransfected GPx1 was used as a normalizer. (D) Western blot showing expression of the MS2 fusion proteins using an anti-HA antibody (upper panel). In the central panel an anti-Flag antibody was used. As a control HeLa cell extract containing a transiently transfected blue fluorescent protein with a C-terminal Flag tag was loaded (EBFP-Flag). Actin was used as a loading control (lower panel). The amount of cell equivalents loaded is indicated at the top of the corresponding lane. (PDF) [file pone.0104391.s004.pdf]

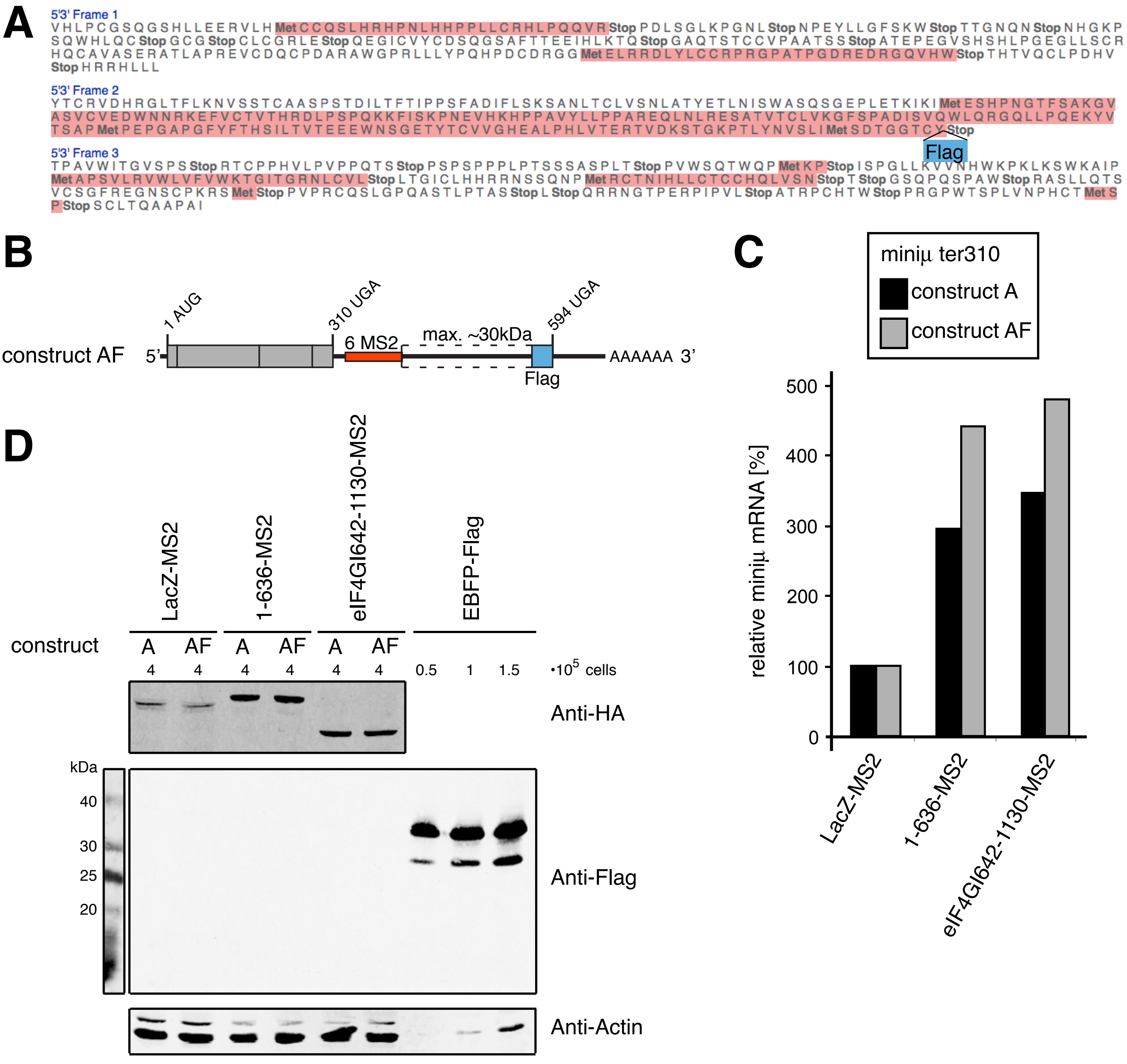
A
5'3' Frame 1
V H L P C G S Q G S H L L E E R V L H Met C C Q S L H R H P N L H H P P L L C R H L P Q Q V R Stop P D L S G L K P G N L Stop N P E Y L L G F S K W Stop T T G N Q N Stop N H G K P
S Q W H L Q C Stop G C G Stop C L C G R L E Stop Q E G I C V Y C D S Q G S A F T T E E I H L K T Q Stop G A Q T S T C C V P A A T S S Stop A T E P E G V S H S H L P G E G L L S C R
H Q C A V A S E R A T L A P R E V C D Q C P D A R A W G P R L L L Y P Q H P D C D R G G Met E L R R D L Y L C C R P R G P A T P G D R E D R G Q V H W Stop T H T V Q C L P D H V
Stop H R R H L L L
5'3' Frame 2
Y T C R V D H R G L T F L K N V S S T C A A S P S T D I L T F T I P P S F A D I F L S K S A N L T C L V S N L A T Y E T L N I S W A S Q S G E P L E T K I K I Met E S H P N G T F S A K G V
A S V C V E D W N N R K E F V C T V T H R D L P S P Q K K F I S K P N E V H K H P P A V Y L L P P A R E Q L N L R E S A T V T C L V K G F S P A D I S V Q W L Q R G Q L L P Q E K Y V
T S A P Met P E P G A P G F Y F T H S I L T V T E E E W N S G E T Y T C V V G H E A L P H L V T E R T V D K S T G K P T L Y N V S L I Met S D T G G T C Y Stop
Flag
5'3' Frame 3
T P A V W I T G V S P S Stop R T C P P H V L P V P P Q T S Stop P S P S P P P L P T S S S A S P L T Stop P V W S Q T W Q P Met K P Stop I S P G L L K V V N H W K P K L K S W K A I P
Met A P S V L R V W L V F V W K T G I T G R N L C V L Stop L T G I C L H H R R N S S Q N P Met R C T N I H L L C T C C H Q L V S N Stop T Stop G S Q P Q S P A W Stop R A S L L Q T S
V C S G F R E G N S C P K R S Met Stop P V P R C Q S L G P Q A S T L P T A S Stop L Stop Q R R N G T P E R P I P V L Stop A T R P C H T W Stop P R G P W T S P L V N P H C T Met S
P Stop S C L T Q A A P A I
B
construct AF
5'
1 AUG
310 UGA
6 MS2
max. ~30kDa
594 UGA
Flag
AAAAAA 3'
C
miniµ ter310
construct A
construct AF
relative miniµ mRNA [%]
0
100
200
300
400
500
LacZ-MS2
1-636-MS2
eIF4GI642-1130-MS2
D
LacZ-MS2
1-636-MS2
eIF4GI642-1130-MS2
EBFP-Flag
construct
A
AF
A
AF
A
AF
4
4
4
4
4
4
0.5
1
1.5
•10^5 cells
Anti-HA
kDa
40
30
25
20
Anti-Flag
Anti-Actin
