# Supplementary material for: Eukaryotic Initiation Factor 4G Suppresses Nonsense-Mediated mRNA Decay by Two Genetically Separable Mechanisms
Source: PLoS One. 2014 Aug 22;9(8):e104391. doi: 10.1371/journal.pone.0104391 (PMC4141738; doi:10.1371/journal.pone.0104391)
Supplement: Table S1 — Proteins identified to interact with the core domain of eIF4GI. Mass spectrometric analysis was carried out on immunoprecipitations of eIF4GI682-1130-MS2. The identified proteins are ordered by Protein Match Score Summation (PMSS). (PDF) [file pone.0104391.s005.pdf]

Table S1

ID	AC	Description	surrogate for abundance (PMSS)
IF4G1_HUMAN	Q04637_ISOFORM_E	Eukaryotic translation initiation factor 4 gamma 1 (eIF-4-gamma 1) (eIF-4G 1) (eIF-4G1) [ISOFORM E]	2680.604
EIF3B_HUMAN	P55884	Eukaryotic translation initiation factor 3 subunit B (eIF3b) (hPrt1)	2292.455
EIF3C_HUMAN	Q99613	Eukaryotic translation initiation factor 3 subunit C (eIF3c)	2259.559
EIF3E_HUMAN	P60228_CHAIN_0	Eukaryotic translation initiation factor 3 subunit E (eIF3e) [CHAIN 0]	1762.456
EIF3A_HUMAN	Q14152_CHAIN_0	Eukaryotic translation initiation factor 3 subunit A (eIF3a) [CHAIN 0]	1627.702
EIF3L_HUMAN	Q9Y262_CHAIN_0	Eukaryotic translation initiation factor 3 subunit L (eIF3l) [CHAIN 0]	1390.801
EIF3D_HUMAN	O15371	Eukaryotic translation initiation factor 3 subunit D (eIF3d)	1156.271
DDX3X_HUMAN	O00571_CHAIN_0	ATP-dependent RNA helicase DDX3X (HLP2) [CHAIN 0]	1074.074
RL4_HUMAN	P36578	60S ribosomal protein L4	1033.03
IF4A1_HUMAN	P60842_CHAIN_0	Eukaryotic initiation factor 4A-I (eIF-4A-I) (eIF4A-I) [CHAIN 0]	1017.252
EIF3I_HUMAN	Q13347	Eukaryotic translation initiation factor 3 subunit I (eIF3i) (TRIP-1)	784.887
EIF3M_HUMAN	Q7L2H7_CHAIN_0	Eukaryotic translation initiation factor 3 subunit M (eIF3m) (hFL-B5) [CHAIN 0]	767.129
DDX21_HUMAN	Q9NR30	Nucleolar RNA helicase 2	751.202
EIF3F_HUMAN	O00303_CHAIN_0	Eukaryotic translation initiation factor 3 subunit F (eIF3f) [CHAIN 0]	750.203
EIF3H_HUMAN	O15372	Eukaryotic translation initiation factor 3 subunit H (eIF3h)	704.473
SRSF1_HUMAN	Q07955_CHAIN_0	Serine/arginine-rich splicing factor 1 (ASF-1) [CHAIN 0]	651.631
EIF3G_HUMAN	O75821_CHAIN_0	Eukaryotic translation initiation factor 3 subunit G (eIF3g) (eIF-3 RNA-binding subunit) [CHAIN 0]	546.95
IF4A2_HUMAN	Q14240	Eukaryotic initiation factor 4A-II (eIF-4A-II) (eIF4A-II)	526.184
IF4G3_HUMAN	O43432	Eukaryotic translation initiation factor 4 gamma 3 (eIF-4-gamma 3) (eIF-4G 3) (eIF4G 3) (eIF4GII)	520.805
EIF3K_HUMAN	Q9UBQ5_CHAIN_0	Eukaryotic translation initiation factor 3 subunit K (eIF3k) [CHAIN 0]	488.15
RL28_HUMAN	P46779_CHAIN_0	60S ribosomal protein L28 [CHAIN 0]	470.985
RL7A_HUMAN	P62424_CHAIN_0	60S ribosomal protein L7a [CHAIN 0]	415.446
EIF3J_HUMAN	O75822_CHAIN_0	Eukaryotic translation initiation factor 3 subunit J (eIF3j) [CHAIN 0]	414.193
RL13_HUMAN	P26373	60S ribosomal protein L13	394.23
SRSF9_HUMAN	Q13242	Serine/arginine-rich splicing factor 9	375.289
RS3A_HUMAN	P61247_CHAIN_0	40S ribosomal protein S3a (Fte-1) [CHAIN 0]	342.561
RL3_HUMAN	P39023_CHAIN_0	60S ribosomal protein L3 (TARBP-B) [CHAIN 0]	315.371
RL30_HUMAN	P62888	60S ribosomal protein L30	289.11
RL18_HUMAN	Q07020_CHAIN_0	60S ribosomal protein L18 [CHAIN 0]	268.12
RL6_HUMAN	Q02878_CHAIN_0	60S ribosomal protein L6 (TaxREB107) [CHAIN 0]	264.086

SRSF3_HUMAN	P84103	Serine/arginine-rich splicing factor 3	261.002
RL10_HUMAN	P27635	60S ribosomal protein L10	243.09
RL17_HUMAN	P18621	60S ribosomal protein L17	226.972
CN166_HUMAN	Q9Y224	UPF0568 protein C14orf166 (CLE)	223.39
DDX1_HUMAN	Q92499	ATP-dependent RNA helicase DDX1 (DBP-RB)	218.934
RS2_HUMAN	P15880	40S ribosomal protein S2	218.084
RS6_HUMAN	P62753	40S ribosomal protein S6	217.578
RS5_HUMAN	P46782	40S ribosomal protein S5, N-terminally processed	216.54
CSK2B_HUMAN	P67870_CHAIN_0	Casein kinase II subunit beta (CK II beta) [CHAIN 0]	216.48
RL15_HUMAN	P61313	60S ribosomal protein L15	215.431
SRSF7_HUMAN	Q16629_ISOFORM_3	Serine/arginine-rich splicing factor 7 [ISOFORM 3]	206.15
