# Supplementary material for: Eukaryotic Initiation Factor 4G Suppresses Nonsense-Mediated mRNA Decay by Two Genetically Separable Mechanisms
Source: PLoS One. 2014 Aug 22;9(8):e104391. doi: 10.1371/journal.pone.0104391 (PMC4141738; doi:10.1371/journal.pone.0104391)
Supplement: Table S2 — Plasmids used in this study. The full name, the oligonucleotides used for cloning and a short description are indicated. The oligonucleotide sequences can be found in Table S3. (PDF) [file pone.0104391.s006.pdf]

Table S2

Name	Oligos	Description
pCMV-MS2-HA		(Eberle et al. 2008)
pCMV-LacZ-MS2-HA		(Eberle et al. 2008)
pCMV-SLBP-MS2-HA	ae80, ae81	
pCMV-PABPC1 (1-636)-MS2-HA		(Eberle et al. 2008)
pCMV-PABPC1 (1-636)-HA		(Eberle et al. 2008)
pCMV-PABPC1 (1-545)-MS2-HA	ae53, ae54, ae55, ae56	PABPC1 without C-terminal domain
pCMV-PABPC1 (180-636)-MS2-HA	ae56, ae62	PABPC1 without RRM1 and RRM2
pCMV-PABPC1 (1-372)-MS2-HA	ae66, om213	RRM1 – RRM4
pCMVPABPC1 (1-372)-HA	om213, rj111	RRM1-4 only with a C-terminal HA-tag
pCMV-PABPC1 (1-279)-MS2-HA	om213, ae67	RRM1 – RRM3
pCMV-PABPC1 (1-636 ΔL)-MS2-HA	om213, ae68, ae70, ae56	PABPC1 lacking the linker domain
pCMV-PABPC1 (1-190)-MS2-HA	om213, ae82	RRM1 – RRM2
pCMV-PABPC1 (180-636 ΔL)-MS2-HA	om213, ae68, ae70, ae56	RRM3 – RRM4 and C-terminal domain (without RRM1-RRM2 and without linker)
pCDNA3.1-HA-MS2-eIF4GIf	ae85, ae86	eIF4GI isoform f
pCMV-eIF4GIf-MS2-HA	rj25, rj26, om214	eIF4GI isoform f
pCMV-eIF4GIe-MS2-HA	rj104, om214	eIF4GI isoform e
pCMV-eIF4GId-MS2-HA	rj105, om214	eIF4GI isoform d
pCMV-eIF4GIb-MS2-HA	rj106, om214	eIF4GI isoform b
pCMV-eIF4GIa-MS2-HA	rj107, om214	eIF4GI isoform a
pCMV-eIF4GIeNT-MS2-HA	rj104, rj34	N-terminus of eIF4GI isoform e (amino acids 41-681 of isoform f)
pCMV-eIF4GI682-1130-MS2-HA	rj35, rj36	Core domain of eIF4GI
pCMV-eIF4GIeΔ682-1079-MS2-HA	rj104, rj38, rj33, om214	eIF4GI isoform e lacking the core domain (lacking amino acids 682-1079 of isoform f)
pCMV-eIF4GI1080-1599-MS2-HA	rj37, om214	C-terminal part of eIF4GI
pCMV-eIF3f-MS2-HA	rj76, rj77	eIF3f
pCMV-eIF3f-HA	rj76, rj77	eIF3f only with a C-terminal HA-tag
pCMV-eIF3h-MS2-HA	rj78, rj79	eIF3h
pCMV-eIF3h-HA	rj78, rj79	eIF3h only with a C-terminal HA-tag
pCMV-HA-MS2-eIF3e	ae164, ae165	eIF3e
pCMV-CTIF-MS2-HA	sr66, sr69	CTIF (CBP80/20-dependent translation initiation factor)
