# Supplementary material for: Eukaryotic Initiation Factor 4G Suppresses Nonsense-Mediated mRNA Decay by Two Genetically Separable Mechanisms
Source: PLoS One. 2014 Aug 22;9(8):e104391. doi: 10.1371/journal.pone.0104391 (PMC4141738; doi:10.1371/journal.pone.0104391)
Supplement: Table S3 — Oligonucleotides used for cloning in this study. (PDF) [file pone.0104391.s007.pdf]

Table S3

Name	Sequence
ae53	5'-TGAAAGGCATGAAGATGCAC-3'
ae54	5'-TAGAAGCCATTGGATCCGGCAAAGGTTCCTGACCTTGT-3'
ae55	5'-CCGGATCCAATGGCTTCTA-3'
ae56	5'-GCTGATTATGATCTAGAGTGC-3'
ae62	5'-AAGGTACCACCATGGAAGCTGAACTTGGAGC-3'
ae66	5'-TTGGATCCGGTTCTTTGCGCTGAGCTAAAGC-3'
ae67	5'-TTGGATCCGGTTTGCGCTTAAGTTCCGTC-3'
ae68	5'-TTCTTTGCGCTGAGCTAAAGC-3'
ae70	5'-GCTTTAGCTCAGCGCAAAGAAACTGCTTCCATGTTGGC-3'
ae80	5'-AAGGTACCATGGCCTGCCGCCCGCGAAG-3'
ae81	5'-ATTAGATCTGGGCTCATGGCTGAGAAGTCTC-3'
ae82	5'-TTGGATCCGGGAATTCTTTTGCCCTAGCTC-3'
ae85	5'-ATGCTAGCCATGGGCTACCCCTACGAC-3'
ae86	5'-TTATTAAGCTTCTCATCCGCGTAGATGCCGGAG-3'
ae164	5'-AAAAGCTTATGGCGGAGTACGACTTG-3'
ae165	5'-ATCTCGAGTCAGTAGAAGCCAGAATCT-3'
om213	5'-GCGTGTACGGTGGGAGGTCTA-3'
om214	5'-GTGTGGGAGGTTTTTTAAAGCAAGT-3'
rj25	5'-ATCGATCGCCACCATGAACAAAGCTCCACAGTCCAC-3'
rj26	5'-ATCCGCGGGGATGGCTTCTAACTTTACTCAGTTCGTTC-3'
rj33	5'-TCTAACAACCAGCTCTTTGCACCTGG-3'
rj34	5'-ATCCGCGGCACGGGTGCTAAGGGTTGTCCG-3'
rj35	5'-ATCGATCGCCACCATGGGGCCCCCAAGGGGTGGGCC-3'
rj36	5'-ATCCGCGGCTACCGCTTGTTGAAGGGCTGAGAAG-3'
rj37	5'-ATCGATCGCCACCATGTCTAACAACCAGCTCTTTGCACCTGG-3'
rj38	5'-CCAGGTGCAAAGAGCTGGTTGTTAGAACGGGTGCTAAGGGTTGTCCG-3'
rj76	5'-CTTAAGCGATCGCCACCATGGCCACACCGGCGGTACC-3'
rj77	5'-CGCCGCCCGCGGCCAGGTTTACAAGTTTTTCATTGAGTGCAATCTGTG-3'
rj78	5'-CTTAAGCGATCGCCACCATGGCGTCCCGCAAGGAAGGTAC-3'
rj79	5'-CGCCGCCCGCGGCGTTGTTGTATTCTTGAAGAGCCTGGGC-3'
rj104	5'-AATTCGACGATCGCCACCATGAACACGCCTTCTCAGCCCCG-3'
rj105	5'-AATTCGACGATCGCCACCATGATGATCCCTTCCCAGATCTCCTAC-3'
rj106	5'-AATTCGACGATCGCCACCATGAACCAGCCACCCCAGATTGC-3'
rj107	5'-AATTCGACGATCGCCACCATGTCTGGGGCCCGCACTGC-3'
rj111	5'-CCATCCCCGCGGCTTCTTTGCGCTGAGCTAAAGC-3'
sr66	5'-GTCTTAGGATCCGCGGCTGTCAGTTTCTGGATG-3'
sr69	5'-ATAGCCGTCGACATGGAAAACTCCTCTGCAG-3'
